# Molecular characterization of bacterial leaf streak resistance in hard winter wheat

**DOI:** 10.7717/peerj.7276

**Published:** 2019-07-15

**Authors:** Sai Mukund Ramakrishnan, Jagdeep Singh Sidhu, Shaukat Ali, Navjot Kaur, Jixiang Wu, Sunish K. Sehgal

**Affiliations:** Department of Agronomy, Horticulture and Plant Science, South Dakota State University, Brookings, SD, USA

**Keywords:** *Triticum aestivum*, BLS (Bacterial leaf streak), GWAS, Resistance, SNPs, *Xanthomonas campestris*, Bacterial diseases, Association mapping, Winter wheat, QTLs

## Abstract

Bacterial leaf streak (BLS) caused by *Xanthomonas campestris pv. translucens* is one of the major bacterial diseases threatening wheat production in the United States Northern Great Plains (NGP) region. It is a sporadic but widespread wheat disease that can cause significant loss in grain yield and quality. Identification and characterization of genomic regions in wheat that confer resistance to BLS will help track resistance genes/QTLs in future wheat breeding. In this study, we evaluated a hard winter wheat association mapping panel (HWWAMP) containing 299 hard winter wheat lines from the US hard winter wheat growing region for their reactions to BLS. We observed a range of BLS responses among the lines, importantly, we identified ten genotypes that showed a resistant reaction both in greenhouse and field evaluation. ­Genome-wide association analysis with 15,990 SNPs was conducted using an exponentially compressed mixed linear model. Five genomic regions (*p* < 0.001) that regulate the resistance to BLS were identified on chromosomes 1AL, 1BS, 3AL, 4AL, and 7AS. The QTLs *Q.bls.sdsu-1AL, Q.bls.sdsu-1BS*, *Q.bls.sdsu-3AL*, *Q.bls.sdsu-4AL*, and *Q.bls.sdsu-7AS* explain a total of 42% of the variation. In silico analysis of sequences in the candidate regions on chromosomes 1AL, 1BS, 3AL, 4AL, and 7AS identified 10, 25, 22, eight, and nine genes, respectively with known plant defense-related functions. Comparative analysis with rice showed two syntenic regions in rice that harbor genes for bacterial leaf streak resistance. The ten BLS resistant genotypes and SNP markers linked to the QTLs identified in our study could facilitate breeding for BLS resistance in winter wheat.

## Introduction

Wheat (*Triticum aestivum L.*) is one of the major cereal crops worldwide. Hard winter wheat contributes 40% (United States Department of Agriculture, 2016) of the total wheat production in the USA; however, it has been challenged by several biotic and abiotic factors, which could limit yield potential and seed quality (Pandey et al., 2017). Bacterial leaf streak (BLS) caused by *Xanthomonas campestris pv. translucens* (*Xct* ), has been emerging as a potential threat to wheat production in the US Great Plains (Kandel et al., 2012) in recent years because most of the current commercial wheat varieties grown in the region appeared to be moderately susceptible or susceptible to BLS (Mueller, 2018). The typical symptoms of BLS are elongated light brown lesions, which are initially distinct, but later may coalesce to cover larger solid areas (Mehta, 2014). BLS can lead to yield losses of up to 40% (Tillman et al., 1999; Mehta, 2014) and can also affect protein content, degrading the grain quality (Duveiller, Van Ginkel & Thijssen, 1992). The pathogen is both residue and seed borne and can disperse long distance via wheat germplasm exchange (Tillman et al., 1999). Chemical control to manage this disease is neither economical nor environmentally safe (Milus & Mirlohi, 1993). Developing BLS resistant cultivars is a more desirable approach in managing BLS in wheat (El Attari et al., 1996). However, several studies showed that resistance against *Xct* was partial in nature in most crops including wheat, barley (*Hordeum vulgare*), and rice (*Oryza sativa*) (Alizadeh et al., 1994; Tillman & Harrison, 1996; Adhikari et al., 2012a; Kandel et al., 2012; Xie et al., 2014), so germplasm screening and characterization still remains a challenge. Therefore, identification and characterization of sources of BLS resistance in wheat would be a vital step in the development of BLS resistant wheat cultivars.

A large assortment of wheat germplasm, including cultivars, breeding lines, and landraces have been previously evaluated for reaction to BLS in the field and/or under the greenhouse conditions (Tillman & Harrison, 1996; Adhikari et al., 2012a; Kandel et al., 2012). Though highly variable reactions among the genotypes were observed, no highly resistant or immune genotypes were found (Tillman & Harrison, 1996; Tillman et al., 1996; Adhikari et al., 2012a; Kandel et al., 2012). Five genes were reported, namely *Bls1, Bls2, Bls3*, *Bls4, and Bls5* from the evaluation of F_3_ populations derived from a set of crosses among five winter wheat parents (Duveiller, Van Ginkel & Thijssen, 1992); however, none of them were genetically mapped. Adhikari et al. (2012a) screened 605 winter wheat accessions from USDA National Small Grain Collection (NSGC) and found only 8.3% of accessions were resistant. Though several germplasm evaluation studies have been performed, only a couple of studies in spring wheat have characterized the genetics of BLS resistance (Adhikari et al., 2012b; Kandel et al., 2015; Wen et al., 2018). Using 566 spring wheat landraces, Adhikari et al. (2012b) identified QTLs for BLS resistance on chromosome 1A, 4A, 4B, and 6A with genetic variation ranging from 1.4% to 2.6%. In another study, Kandel et al. (2015) reported QTLs on chromosomes 1B, 2A, 3B, and 6A in a multifamily population of spring wheat. The significant SSR markers in this study explained a variation ranging from 0.5% to 23%. A recent study by Wen et al. (2018) showed that mapped BLS resistance gene in Triticale (X *Triticosecale* Wittmack) was harbored on chromosome 5R. BLS being a major disease of rice as well, some major genes and several QTLs for BLS resistance have been also reported in rice (Tang et al., 2000; Chen et al., 2006; He et al., 2012). However, to our knowledge, there have been no reports on molecular genetic characterization of BLS resistance in winter wheat germplasm.

Quantitative traits like BLS can generally be dissected with tools such as linkage analysis or association mapping (AM), where AM exploits historical recombination in the existing set of lines from natural collections, breeding populations, or varieties (Zhu et al., 2008). Thus, with the availability of high-throughput marker technologies, genome-wide association studies (GWAS) have emerged as an effective strategy to capture QTLs and novel alleles particularly in genetically diverse germplasm or wild relatives (Zhao et al., 2011; Begum et al., 2015) as compared to parental populations (Buckler & Thornsberry, 2002). With this approach, no prior information on the marker-trait associations are required and multiple loci can be conveniently identified (Bordes et al., 2011). Several robust statistical methods and tools including mixed linear models (Yu et al., 2006; Zhang et al., 2010), Bayesian clustering (Pritchard & Rosenberg, 1999; Pritchard, Stephens & Donnelly, 2000), principal component analysis (PCA) (Price et al., 2006) have been developed and used to enhance our understanding of complex traits in animal and plant genetic systems. With above-mentioned methods, several studies have characterized resistance to stem rust (Bariana et al., 2001; Yu et al., 2011; Letta et al., 2014; Zhang et al., 2014; Muleta et al., 2017), solid stem for sawfly (Varella et al., 2015), leaf rust (Singh, Nelson & Sorrells, 2000; William et al., 2006; Turner et al., 2017), spot blotch (Ayana et al., 2018) and several other diseases (Arif et al., 2012; Bentley et al., 2014; Dababat et al., 2016) in wheat as well as other crops like rice (Huang et al., 2010; McCouch et al., 2016; Raboin et al., 2016) and maize (*Zea* mays) (Wang et al., 2016; Liu et al., 2017; Xiao et al., 2017). In the same manner, GWAS provides a potential to identify loci controlling BLS resistance in wheat and can facilitate wheat improvement through marker-assisted pyramiding of favorable alleles and backcross breeding. Therefore, the objectives of this study were to (1) evaluate winter wheat varieties and breeding lines from eight major hard winter wheat breeding programs in the United States to identify sources of BLS resistance and (2) identify genomic region conferring BLS resistance and develop SNP markers to enable marker-assisted selection.

## Material & Methods

### Plant materials

In the present study, we used a hard winter wheat association mapping panel (HWWAMP) of 299 winter wheat accessions developed under the USDA TCAP project (Guttieri et al., 2015). The source and geographic diversity of these 299 HWW accessions are shown in Fig. S1. Additional physiological and agronomic data about the HWWAMP accessions is available in T3/Wheat database (https://triticeaetoolbox.org/wheat/pedigree/pedigree_info.php). The experiments were conducted both in the greenhouse (GH) for controlled conditions (2015–2016) and in the field at the South Dakota State University Agriculture Experimental Station at Brookings, SD (2016–2017).

### Genotyping

The HWWAMP has been genotyped using the wheat *Infinium* 90K *iSelect* array (Illumina Inc. San Diego, CA) under the USDA-TCAP (Guttieri et al., 2015) and the genotype data were obtained from T3 Toolbox (https://triticeaetoolbox.org/wheat/). To avoid spurious marker-trait associations, SNP markers with minimum allele frequency (MAF) <0.05 and >10% missing data were excluded from further analyses. The genetic positions of the selected SNP markers from the wheat *Infinium* 90K *iSelect* were obtained from the consensus map of 46,977 SNPs (Wang et al., 2014). The SNPs flanking sequences were mapped using BLASTN to wheat RefSeq v1.0 assembly (International Wheat Genome Sequencing Consortium, 2018) to identify the physical locations of the mapped SNPs.

### Phenotypic evaluation and statistical analysis

#### Planting and experimental design

The entire study consists of two greenhouse experiments (GH1 and GH2 in 2016 and 2017, respectively) and one field experiment (2017) constituting of two replications each. Both in the field and the greenhouse, spring wheat cv. Briggs (susceptible check: SC1) and germplasm accession SD1001 (pedigree: PFAU/MILAN//TROST, susceptible check: SC2); SD52 (pedigree: CNO79//PF70354/MUS/3/PASTOR/4/BAV92*2/5/HAR311, resistant check: RC1) were included in the study as susceptible and moderately resistant checks, respectively.

For greenhouse experiments, three seeds of each accession were planted in the cones which were 3.8 cm in diameter and 20 cm in height (Stuewe and Sons, Inc., Corvallis, OR). To the soil in each container, 2.5 g of multicote slow release commercial fertilizer with a 14–14–16 Nitrogen Phosphorous and Potassium composition (Sungro Horticulture Distribution Inc. Agawam, MA, USA) was applied at the time of planting. Each cone consisted of three plants per replication. Each cone was considered as an experimental unit, and the third leaf of each plant was regarded as a sampling unit. Both greenhouse experiments were performed under controlled temperature: 30/18 °C diurnal cycle (day/night) with a 16 h photoperiod and relative humidity (>85%) (Silva et al., 2010) in a randomized complete block design. In the field experiment, the 299 HWWAMP accessions were planted in ∼90 cm long single row plots (40 seeds per row) with a plot to plot distance of 19.5 cm. In total there were two blocks (1rep per block) along with resistant and susceptible checks. The experimental unit in the field was 10 flag leaves from each plot. Accessions were randomized in each block and for better representation, three plots of checks were planted in each block.

#### Inoculum, infiltration and disease assessment

A highly virulent strain *isolate Xct-017* of *X. campestris pv. translucens*, was used in this study. A fresh culture of the isolate *Xct-017* was initiated from −80° C freezer by streaking on a Kim-Goepfert (KG) agar media (agar = 20 g; magnesium sulphate = 1.5 g; proteose peptone = 20 g; potassium phosphate = 1.5 g) (HiMedia Labs Inc. Mumbai, India). The bacterial cells were obtained from a two day old culture by adding 20–25 ml of distilled water into each plate and scraping the surface using a flamed microscope slide. The inoculum density was adjusted to 3 × 10^8^ colonies forming unit ml^−1^ using a turbidometer (Biolog Inc., Hayward, CA, USA). In the greenhouse, the infiltration was performed by injecting 10 to 15 µL of inoculum into a fully expanded third leaf using a needleless disposable syringe (Faris et al., 1996). The infiltrated areas were marked by a non-toxic sharpie, permanent marker and the plants were placed in trays with water. These plants were kept in a mist chamber for 24 h post-infiltration to enhance the infection process. The mist chamber was built in house and a humidifier (Vicks Filter-free cool mist humidifier-V4600) was installed for moisture. With the help of a timer, mist from the humidifier was released every 15 min for a time span of 4 min. After 24 h the plants were moved to a growth chamber (Conviron A1000 growth chamber). In the field, at third leaf stage, plants were inoculated using a powered backpack mist blower (Solo 451; Solo, Sindelfingen, Germany) with a fixed pressure. A 3 g of Carborundum (Fischer Scientific) was added to 1 liter of inoculum (1 × 10^8^ cfu/ml) to create non-lethal wounds on the leaves.

#### Disease rating

BLS is a very complex disease and requires stringent and highly accurate phenotyping. We rated the genotypes after 14 days of infiltration or inoculations. In the greenhouse environment (a controlled environment), we applied a disease rating scale of 1–5. The length of the initial infiltration area (L0) was recorded and the length of the final infected area (L14) was recorded. The difference in the length (L14-L0) was calculated and used to determine the rating level for each plant (Fig. S2) as following: category 1 (resistant, R): 0–0.49 cm increase; category 2 (moderately resistant, MR): 0.5–0.9 cm increase; category 3 (moderately susceptible, MS): 1–1.49 cm increase; category 4 (susceptible, S): 1.5–1.99 cm increase; and category 5 (highly susceptible, HS): ≥ 2 cm. In field evaluation, we rated the plants as percent leaf area necrotic ; 1: 0–20%, 2: 21–40%, 3: 41–50%, 4: 51–70% at the soft dough stage (Feeks 11.2) and 5: >70%. The statistical parameters including mean, frequency distribution, analysis of variance (ANOVA) were calculated using a statistical program R (De Mendiburu Delgado, 2009; Team, 2014). Broad sense heritability (H^2^) for field and greenhouse experiments was estimated separately using Eqs. (1) and (2) respectively.

(1) \begin{eqnarray*}& & {H}^{2}= \frac{{\sigma }_{G}^{2}}{{\sigma }_{G}^{2}+{\sigma }_{e}^{2}/r} \end{eqnarray*}

(2) \begin{eqnarray*}& & {H}^{2}= \frac{{\sigma }_{G}^{2}}{{\sigma }_{G}^{2}+{\sigma }_{E}^{2}/n+{\sigma }_{{G}^{\ast }E}^{2}/nr} \end{eqnarray*}

\begin{eqnarray*}& & {\sigma }_{G}^{2}=genotype, {\sigma }_{e}^{2}=error, \end{eqnarray*}

\begin{eqnarray*}& & {\sigma }_{E}^{2}=experiment, {\sigma }_{{G}^{\ast }E}^{2}=genotype\ast experiment, \end{eqnarray*}

\begin{eqnarray*}& & r=number~of~replications, \mathrm{and}~n=number~of~experiments \end{eqnarray*}

Combined best linear unbiased estimator (BLUE) values for GH and field data were calculated using Eq. (3) and employing META-R (Vargas et al., 2013).

(3) \begin{eqnarray*}{\mathrm{Y }}_{ijk}=\mu +{\mathrm{Gen}}_{\mathrm{i}}+{\mathrm{Exp}}_{\mathrm{j}}+{\mathrm{Rep}}_{\mathrm{k}}({\mathrm{Exp}}_{\mathrm{j}})+{\mathrm{Gen}}_{\mathrm{i}}\times {\mathrm{Exp}}_{\mathrm{j}}+{\mathrm{e}}_{\mathrm{ijk}}\end{eqnarray*}

where Y_ijk_ is the BLS score, µ is the mean effect, Gen_i_ is the effect of the ith genotype, Rep_k_ is the effect of the kth replicate, Exp_j_ effect of jth experiment, and e_ijk_ is the error.

### Association mapping analysis

GWAS analysis for BLS resistance was performed with GAPIT (Tang et al., 2016) using 299 hard winter wheat accessions and 15,990 SNPs with MAF >5%. The marker properties, Linkage disequilibrium (LD), principal component (PC) matrix, hierarchical clustering, and Q+K mixed model (Brbaklic et al., 2013) were estimated to account for structure (Q) and kinship (K) in GAPIT. Kinship was estimated using default VanRaden algorithm. Data from the appropriate number of PCs and the allele sharing similarity matrix accounting for Q and K were fit into the linear model to associate numeric SNP genotypes with ordinal phenotypes. The “*Q*” parameter was accounted by the PC and the PC scores were used in the model as covariates. Additive genetic model in GAPIT was used to perform association tests. The negative log_10_ (*P*-value) conversion was used on all calculated *P*-values. QQ (quantile–quantile) plots assuming a uniform distribution of p-values under the null hypothesis of no QTLs were used to see inflation in the results and evaluate the models. Both enriched compressed mixed linear model (*ECMLM*) and compressed mixed linear model (CMLM) accounted well for population structure and kinship and seeing better statistical power of ECMLM over CMLM (Li et al., 2014), we selected ECMLM to analyze the marker-trait associations (MTA).

The ECMLM model is based on a unified-mixed model (Yu et al., 2006; Li et al., 2014) and described by Henderson’s notation.

(4) \begin{eqnarray*}y=X\beta +Zu+e\end{eqnarray*}

where *y* represents a vector of the phenotype, *β* represents the unknown fixed effects like population structure and marker effects, *u* represents the unknown polygenic effects or random effects like kinship, *X* and *Z* are the incidence matrices for *β* and *u*, respectively and e represents the error.

These results were also validated using the MLM (Mixed Linear Model) (Zhang et al., 2010) in TASSEL. To declare the significant marker–trait associations (MTAs), we considered a threshold *p*-value of −log10 (p) ≥3. The proportion of the phenotypic variation (*R*^2^) was calculated using GAPIT

The QTLs were also validated using a 5-fold validation (Sidhu et al., 2019), briefly, the total set was divided into five subsets of equal size selected randomly without replacement. Out of these five subsets, four were used for marker-trait association analysis and the last set was used to cross-validate the significant markers using *t*-test among different alleles of each marker and 1000 iterations of this cross-validation were performed. We identified MTAs for: (1) BLS reaction under controlled greenhouse conditions (average of GH1 and GH2), (2) BLS reaction under field conditions, (3) best linear unbiased estimates (BLUEs) from combining greenhouse (average of GH1 and GH2) and field data to identify consistent QTLs for BLS resistance in wheat.

### Haplotype analysis

All the significant markers in the candidate region were selected and arranged according to their physical position on IWGSC Refseqv1.1. Then, the significant SNPs in each of the candidate region were tested for Hardy–Weinberg equilibrium using the Java program –Haploview 4.2. (Barrett et al., 2005). Linkage Disequilibrium was estimated as D′ and represented in the figure as D′/LOD. Haplotype blocks were decided based on the four Gamete rule in Haploview.

### In-silico annotation of candidate regions

To identify candidate genes conferring BLS resistance, the regions flanking the critical SNPs (SNP with highest −log_10_ (*P*-value)) were extracted from the Infinium iSELECT 90K (Cavanagh et al., 2015). The SNPs from each candidate regions were BLASTN searched against the IWGSC wheat RefSeq v1.0 to extract the target QTL regions from the CS pseudomolecule (International Wheat Genome Sequencing Consortium, 2018). The target regions were repeat masked with RepeatMasker (http://www.repeatmasker.org/) to identify the coding (CDS) and low-copy sequences. The CDS sequences were then annotated by BLASTX against the wheat protein database (Altschul et al., 1990; Duncan et al., 2017) and the putative functions of the proteins were identified using Pfam database (Bateman et al., 2004). The genes with plant defense-related functions were identified as candidate genes conferring BLS resistance in wheat.

### Comparative analysis with rice

For comparative analysis, we mapped the candidate SNPs on the IWGSC wheat RefSeqv1.0 (International Wheat Genome Sequencing Consortium, 2018). The wheat genome sequence was repeat masked with RepeatMasker (http://www.repeatmasker.org/) using mipsREdat 9.3p Poaceae TEs repeat database (Nussbaumer et al., 2012) to identify low copy regions. BLASTN against wheat CDS databases was then performed using standalone BLAST API onto a Linux based high-performance cluster (Altschul et al., 1990) to identify coding sequences in the candidate region. The synteny in the candidate region was then performed by BLASTN search against rice genome CDS (Kawahara et al., 2013).

## Results

### Phenotypic analysis

**Figure 1:**
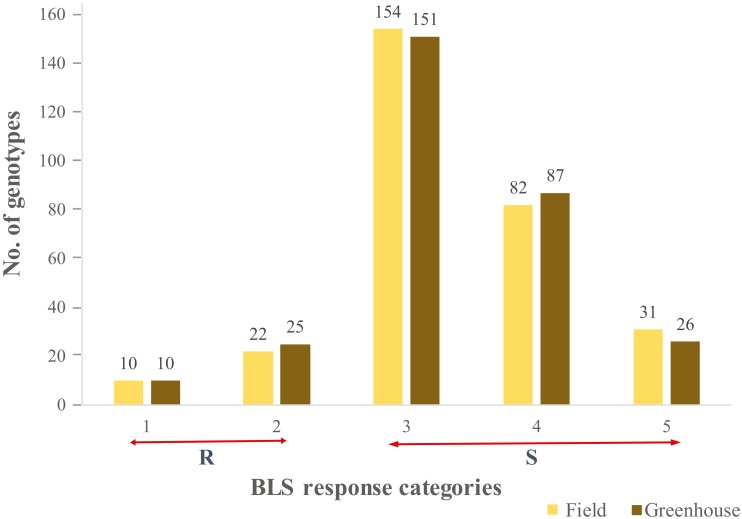
Distribution of the 299 genotypes of hard winter wheat association mapping panel (HWWAMP) into the 5 categories based on response to Bacterial leaf streak (BLS). The bars represent the mean response of the genotypes to BLS in both the greenhouse and field (1-Resistant, 2-Moderatly Resistant, 3-Moderately Susceptible, 4-Susceptible, and 5-Highly Susceptible).

A continuous range of response to BLS infiltration was observed in HWWAMP accessions. As expected, susceptible checks (SC1, SC2), and moderately resistant check MRC1 exhibited susceptible and moderately resistant reactions, respectively. In the greenhouse (GH) experiments, the mean disease score was 3.2 and 3.3 for GH1 and GH2, respectively (Table S1). The correlation coefficient between the two GH experiments was 0.97. In the field, mean disease score was 3.3 (Table S1). Of the 299 genotypes, 10 genotypes (3.0%) exhibited a consistent resistant reaction to BLS in greenhouse and field experiments (category 1), whereas another 25 (8.0%) and 22 (7.3%) accessions demonstrated a moderate resistance response (category 2) in greenhouse and field, respectively. We observed 151 (50.5%) and 154 (51.5%) lines had a moderately susceptible reaction (category 3), whereas 87 (29%) and 82 (27.7%) lines showed susceptible (category 4) response in greenhouse experiments and field, respectively. Another 26 (8.6%) and 31 (10.3%) lines showed a highly susceptible reaction (category 5) in the greenhouse and field experiments, respectively (Fig. 1). Analysis of variance (ANOVA) for BLS scores revealed significant differences among genotypes in the greenhouse (Table S2) as well as field conditions (Table S3). The broad sense heritability (H^2^) in the greenhouse and field experiment was 0.97 and 0.75, respectively.

### Genotyping

The *Infinium* 90 K *iSelect* genotype data for 299 hard winter wheat accessions was obtained (T3 database) and constituted 21,500 polymorphic SNP markers. Of these, 5,510 markers had MAFs of <0.05 or greater than 10% missing genotypic data or missing map positions and therefore they were eliminated from further analysis, resulting in a total of 15,990 markers that were utilized for association analysis. The majority of SNP markers were located across wheat A and B genomes (40% and 50%, respectively) while the D genome had comparatively a lower coverage (10%). The highest number of SNP markers were located on chromosome 5B followed by 1B, 6B, and 2B (Fig. S3). The average number of SNP markers per wheat chromosome was 750.

### Marker-trait association (MTA) analysis for BLS response

**Figure 2:**
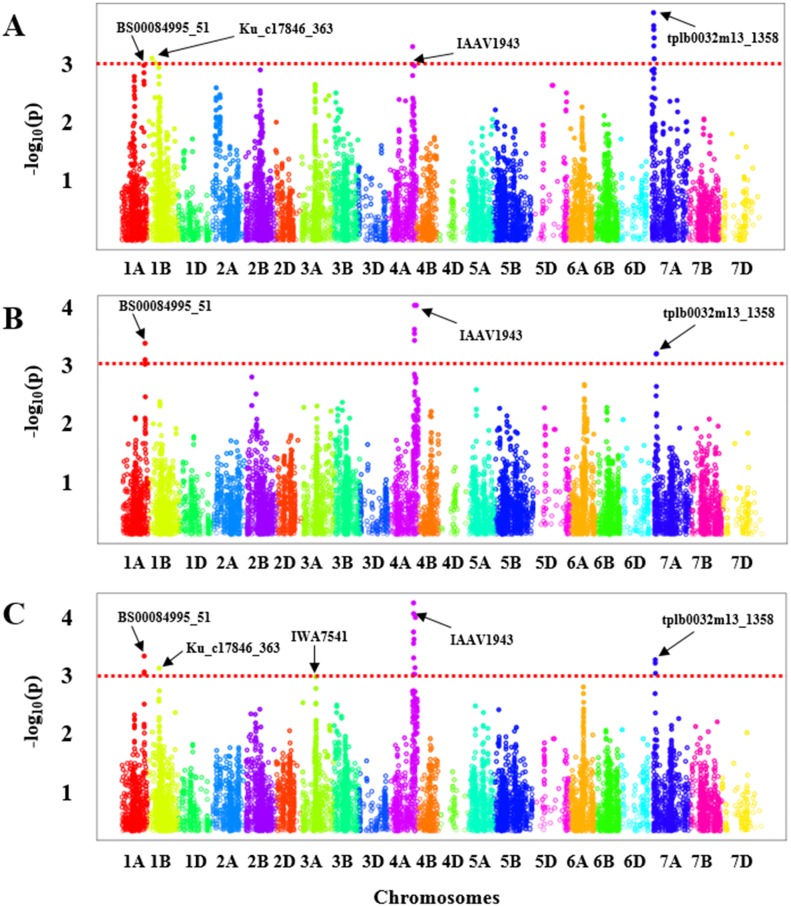
Manhattan plot with the −log_10_ (*P*-value) of all SNPs used in GWAS for bacterial leaf streak (BLS) on 299 genotypes of the Hard winter wheat association mapping panel (HWWAMP) using enriched compressed mixed linear model (ECMLM). (A) Greenhouse experiments, (B) Field experiment and (C) best linear unbiased estimator (BLUE) values. The red dotted line in the figure shows the threshold of −log_10_ (*p*-value) = 3 and all the significantly associated SNP markers are above the red line.

GWAS was carried out for each experiment (greenhouse experiments combined: Fig. 2A and Field Experiment: Fig. 2B) separately. To ascertain the consensus QTLs we calculated BLUE values for all genotypes to perform marker-trait associations (Fig. 2C). In the greenhouse experiments, a total of four significant regions were obtained with one each on chromosome 1A, 1B, 4A, and 7A (Fig. 2A, Table S4) that showed an association with BLS resistance, whereas from the field evaluation, we only identified three significant genomic regions on the chromosomes 1A, 4A, and 7A (Fig. 2B, Table S4) in HWWAMP. When BLUE values were used for GWAS analysis, we identified a total of five genomic regions associated with BLS resistance located on chromosomes 1A, 1B, 3A, 4A, and 7A (Fig. 2C, Table S4). A total of 20 significant MTAs across these five chromosomes were identified for BLS resistance (*p* < 0.001) in the HWWAMP. These five genomic regions were further validated using MLM models in TASSEL and SUPER algorithms of GAPIT to further ascertain consensus and significance. All five QTLs, *Q.bls.sdsu-1AL, Q.bls.sdsu-1BS*, *Q.bls.sdsu-3AL*, *Q.bls.sdsu-4AL,* and *Q.bls.sdsu-7AS* were detected by all the algorithms in TASSEL and GAPIT (Table 1). These genomic regions showed significant association (*P*-value <0.001) with 3, 2, 1, 11, and 3 SNPs, respectively. The most significant SNPs linked to *Q.bls.sdsu-1AL, Q.bls.sdsu-1BS*, *Q.bls.sdsu-3AL*, *Q.bls.sdsu-4AL* and *Q.bls.sdsu-7AS* were BS00084995_51, Ku_c17846_363, IWA7541, IAAV1943, and tplb0032m13_1358, respectively (Table 1). The five QTLs *Q.bls.sdsu-1AL* (8.3%)*, Q.bls.sdsu-1BS* (8.5%), *Q.bls.sdsu-3AL* (7.9%), *Q.bls.sdsu-4AL* (8.8%), and *Q.bls.sdsu-7AS* (9.3%) explained 42% of the total variation in BLS (Table 1). The flanking SNPs spanned the QTLs regions to an estimated 2.05 cM (1AL), 3.5 cM (1BS), 0.013 cM (3AL), 2.09 cM (4AL), and 0.15 cM (7AL), corresponding to approximately 4.4 Mb, 3.1 Mb, 46.2 Mb, 3.3 Mb, and 3.0 Mb, respectively.

After validating the QTLs with several algorithms, we further ascertained the significance of the obtained SNP markers in each genomic region by performing a 5-fold cross-validation. The HWWAMP was randomly split into five subsets, four were used for the marker-trait association and the last set was used to cross-validate the significant markers. The cross-validation confirmed that all five SNPs BS00084995_51 (1A), Ku_c17846_363 (1B), IWA7541 (3A), IAAV1943 (4A), and tplb0032m13_1358 (7A) were significantly associated with BLS resistance as the *P*-value was <0.001 (Table 1).

### Comparative analysis of the QTL regions in rice and wheat

BLS is a threatening disease in rice and several QTLs for bacterial leaf streak resistance have been reported in rice (Tang et al., 2000). Identifying a syntenic relationship of QTLs identified in wheat with rice could be another means to validate the QTLs identified in our study and therefore, we performed a synteny analysis of wheat CDS sequences from the candidate regions against the rice CDS sequences associated with BLS (Matsumoto et al., 2005). Of the five QTLs identified in our study, two QTLs *Q.bls.sdsu-3AL* and *Q.bls.sdsu-4AL* could potentially be syntenic to known BLS resistance QTLs *qBlsr1, and qBlsr3d* on chromosomes 1R, and 3R in rice, respectively (Fig. S4) (Tang et al., 2000; He et al., 2012). The QTLs mapped in our study were also mapped near the RFLP/AFLP markers reported in rice. *qBlsr1* in rice is mapped on chromosome 1 and linked to wheat RFLP probe *Xpsr* 116 (Tang et al., 2000), we found *Q.bls.sdsu-3AL* (IWA4571) to be in the same region. However, very precise comparison for QTLs *Q.bls.sdsu-4AL* could not be made as the sequences for RFLP/AFLP markers linked to *qBlsr3d* in rice are not available and therefore we had to rely on proximal marker *C63A* that also mapped proximal to *Q.bls.sdsu-1AL* in wheat.

**Table 1:** Summary of significant SNPs linked to QTLs for Bacterial leaf streak (BLS) resistance detected from the best linear unbiased (BLUE) values of greenhouse and field evaluations.

QTL	Marker	cM^a^	Mb^b^	R Allele^c^	S Allele^d^	*p*-value	*R*^2^%	Additive effect	*T*-test^e^
*Q.bls.sdsu-1AL*	BS00084995_51	139.74	580.42	T	G	1.16e^−3^	8.3	−0.20	9.82e^−6^
*Q.bls.sdsu-1BS*	Ku_c17846_363	24.54	9.54	C	T	8.91e^−4^	8.5	−0.26	1.10e^−3^
*Q.bls.sdsu-3AL*	IWA7541	89.48	533.07	G	A	1.31e^−3^	7.9	−0.22	5.74e^−3^
*Q.bls.sdsu-4AL*	IAAV1943	144.37	726.44	T	C	1.03e^−4^	8.8	−0.21	5.66e^−4^
*Q.bls.sdsu-7AS*	tplb0032m13_1358	43.47	10.25	T	C	2.53e^−4^	9.3	−0.22	2.21e^−3^

**Notes.**

a The cM (centimorgan) location is from Wang et al. (2014).

b The SNP position (Mb) was identified by BLAST on IWGSC RefSeqv1.0 (International Wheat Genome Sequencing Consortium, 2018).

c Resistant allele.

d Susceptible allele.

e *p*-value obtained from the 5-fold cross-validation.

### Identification of candidate genes and putative functions

In all five QTL regions, a total of 588 genes with known functions were predicted. Among all, 74 genes were predicted to belong to one of the following disease resistance families: LRR (Leucine-rich repeat), NB-ARC (nucleotide- binding, Apaf-1, Rproteins, and Ced-4), NPR1_like_C (NPR1/NIM1 like defense protein C terminal), and Pkinase (Protein kinase domain) (Table S5). In a 4.36 Mb region spanning *Q.bls.sdsu-1AL* we predicted 61 genes in total, of which ten genes have known pathogen resistance related function. In particular, three had NB-ARC domain, whereas, another three genes had receptor-like protein kinase domains. The *Q.bls.sdsu-1BS* region was flanked by BS00066271_51 and BS00100994_51, from 6.87 Mb to 9.94 Mb on the short arm of chromosome 1B and a total of 73 genes including 25 having a likely disease resistance function. Specifically, 13 genes had NB-ARC domain, seven genes had Pkinase domain, three genes were predicted as a wall-associated kinase (WAK) and another two genes had LRR domain. The QTL *Q.bls.sdsu-3AL* extends from 487.45 Mb (wsnp_Ex_rep_c69577_68526990) to 533.63 Mb (Excalibur_rep_c67269_496) on the long arm of chromosome 3A. This region is the longest one with 46.2 Mb and we found 348 genes in total, including 22 genes predicted to have a pathogen resistance function. These regions include three genes with NB-ARC, six genes with LRR and seven with PKinase domains. Another QTL, *Q.bls.sdsu-4AL* spanning the 3.3Mb region (723.81 Mb to 727.18 Mb) harbored 46 genes with known functions including eight related to pathogen resistance, with six genes having an NB-ARC type domain and others having Pkinase or LRR domains. The fifth QTL, *Q.bls.sdsu-7AS* was delimited to 3Mb long region flanked by SNPs RAC875_c41169_68 and Tdurum_contig46954_406. In this region, we discovered a total of 60 genes including nine genes with a potential role in disease resistance, in specific, we identified, one gene from NB-ARC family, one from receptor-like protein kinase family, another two genes had LRR domain and five had Pkinase domain.

**Figure 3:**
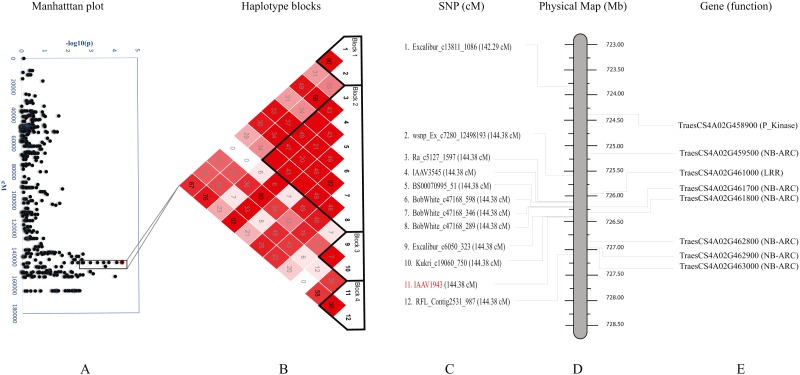
Haplotype analysis and detailed gene annotation of the *Q.bls.sdsu- 4AL* region conferring resistance to Bacterial leaf streak (BLS). (A) Manhattan plot with –log10(*p*) value on *y*-axis and marker position (cM) on the *x*-axis. Significant region (*Q.bls.sdsu-4AL*) is encompassed in a black rectangle containing 12 markers. (B) Haplotype blocks generated using haploview. (C) SNPs with corresponding cM positions in parenthesis along with the most significant marker (red). (D) Physical location of SNP in Mb. (E) Predicted CDS genes with known defense-related function in the *Q.bls.sdsu-4AL* region.

**Figure 4:**
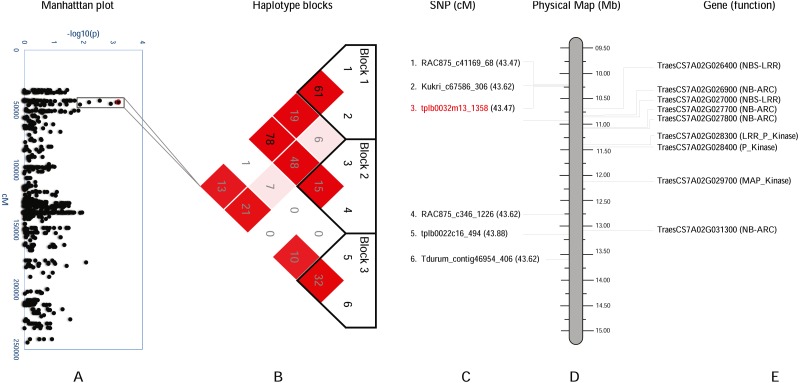
Haplotype analysis and detailed gene annotation of the *Q.bls.sdsu- 7AS* region conferring resistance to Bacterial leaf streak (BLS). (A) Manhattan plot with −log10(p) value on *y*-axis and marker position (cM) on the *x*-axis. Significant region (*Q.bls.sdsu- 7AS*) is encompassed in a black rectangle containing 6 markers. (B) Haplotype blocks generated using haploview. (C) SNPs with corresponding cM positions in parenthesis along with the most significant marker (red). (D) Physical location of SNP in Mb. (E) Predicted CDS genes with known defense-related function in the *Q.bls.sdsu- 7AS* region.

We further studied haplotypes for two QTL*s Q.bls.sdsu-4AL* (Fig. 3) and *Q.bls.sdsu-7AS* (Fig. 4) as these explained the largest phenotypic variation in our study. In *Q.bls.sdsu-4AL* region, we found four haplotype blocks, marker IAAV1943 (most significant SNP) was in block 4, approximately 1Mb long (Fig. 3). In block 4, four genes code pathogenesis-related (PR) NB-ARC proteins (Table S5).

In the *Q.bls.sdsu-7AS* region, we discovered three haplotype blocks, SNP tplb0032m13_1358 (most significant) is found in block 2, which is approximately 2.5Mb long region (Fig. 4). In block 2 a total of eight pathogenesis-related (PR) protein-coding genes were identified, out of which three code proteins with P-Kinase domain, another two code for NBS-LRR and rest three code for NB-ARC (Table S5).

## Discussion

BLS impacts many crop species, including wheat, Triticale, rice, and Brassica (El Attari et al., 1996; Tang et al., 2000; Wechter et al., 2014; Wen et al., 2018). Development of disease resistant cultivars has been considered as one of the most effective management strategies in the absence of effective chemical control (Milus & Mirlohi, 1995). However, the progress in the development of BLS resistant cultivars is hampered by its complex inheritance, less understanding of the resistance mechanism, and unavailability of desirable molecular markers. Some major genes and several QTLs for BLS resistance have been reported in rice (Tang et al., 2000). Recently, Wen et al. (2018) reported a major QTL on 5R of Triticale. In an earlier study, Duveiller, Van Ginkel & Thijssen (1992) developed complete series of crosses between five wheat parents and in F_3_ generations attested that five genes (*Bls1*, *Bls2*, *Bls3*, *Bls4*, and *Bls5*) were involved in the resistance to BLS. These studies (Tillman & Harrison, 1996; Tillman et al., 1996; Adhikari et al., 2012b; Kandel et al., 2012) showed that resistance to BLS in wheat is partial. Most of these genetic studies were performed in spring wheat (Tillman & Harrison, 1996; Adhikari et al., 2012b; Kandel et al., 2015) with only a couple of studies on the characterization of BLS resistance in winter wheat (Tillman & Harrison, 1996). To our knowledge, this is the first study that characterizes QTLs for BLS resistance in winter wheat. In the present study, we have evaluated HWWAMP of 299 winter wheat accessions for their response to BLS in field and greenhouse conditions. The frequency distributions observed for both greenhouse and field BLS was slightly skewed towards susceptibility as expected since most of the germplasm in wheat is known to be moderate to highly susceptible to BLS, further suggesting that BLS is a quantitatively inherited trait in wheat (Duveiller, Van Ginkel & Thijssen, 1992; Tillman & Harrison, 1996; Adhikari et al., 2012b), which is similar to rice (Tang et al., 2000; Tran et al., 2008; Poulin et al., 2014).

The mean disease incidence in the greenhouse was slightly lower than in the field, which could be attributed to the differences in the infiltration and inoculation methods or environmental conditions. We identified ten accessions (3.0%) showing resistance to BLS (Table 2). Many of the lines showing resistance to BLS have a pedigree or a selection involving the cultivar ‘Scout’. The cv. Scout was released in 1963 by the University of Nebraska at Lincoln and later a selection was made for earliness and better quality. This selection was released as Scout 66 (1967). The cultivar Scout 66 is also one of the 10 resistant genotypes of the HWWAMP. This indicates that Scout could be one of the major sources of BLS resistance in the HWWAMP. In addition, Scout has been reported to be resistant to Hessian fly, tan spot, and soil-borne diseases. It is moderately resistant to leaf rust and susceptible to stem rust and stripe rust (Fall Seed guide –UNL, 2016). In this study, a large number of genotypes showed a moderately susceptible reaction (152) to susceptible reaction (113) demonstrating the importance of resistant germplasm. Many moderately susceptible to susceptible genotypes showed cv. ‘2180′ in their pedigree. The cultivar 2180 (PI 532912) was released by Kansas State University in 1989 and it shows a moderately susceptible reaction to BLS in our study. It is possible that cv. 2180 is a source of susceptibility to bacterial leaf streak in our panel.

**Table 2:** List of ten genotypes from the Hard winter wheat association mapping panel (HWWAMP) that showed resistance reaction against bacterial leaf streak (BLS).

Genotype	Pedigree	PI
EAGLE (KS: 1970)	Selection from Scout	Cltr15068
		
GOODSTREAK (NE: 2002)	Len//Butte/ND526/6/Agent/3/ND441//Waldron/Bluebird/4/Butte/5/ Len/7/KS88H164 /8/NE89646	PI632434
		
NE04490	NE95589/3/Abilene/Norkan//Rawhide/4/Abilene/Arapahoe	–
OK05723W	SWM866442/Betty	–
OK1068112	Farmec/Jagalene	–
ROBIDOUX (NE: 2010)	Odesskaya P/Cody//Pavon 76/3* Scout 66/3/Wahoo	PI659690
		
SCOUT66 (NE: 1967)	Nebred//Hope/Turkey/3/ Cheyenne/Ponca	CI13996
		
TX07A001420	U1254-1-5-2-1/ TX81V6582// Desconocido	–
VISTA (NE: 1992)	Warrior//Atlas66/Comanche/3/Comanche/Ottawa/5/Ponca/2* Cheyenne/3/Illinois No. 1//2* Chinese Spring /T. timopheevii/4/Cheyenne/Tenmarq//Mediterranean/Hope/3/ Sando60/6/Centurk/Brule	PI562653
		
WENDY (SD: 2004)	Gent/Siouxland//Abilene	PI638521

Using GWAS for BLS resistance we identified five QTLs conferring resistance to BLS in winter wheat. Combined greenhouse and field data provides more confidence behind these QTLs. The five QTLs on five different chromosomes namely, 1AL (*Q.bls.sdsu-1AL*), 1BS (*Q.bls.sdsu-1BS*), 3AL (*Q.bls.sdsu-3AL*), 4AL (*Q.bls.sdsu-4AL*), and 7AS (*Q.bls.sdsu-7AS*) explained 8.3%, 8.5%, 7.9%, 8.8%, and 9.3% of the variation, respectively. They collectively accounted for 42.3% of the total variation. Two QTLs, *Q.bls.sdsu-1AL* and *Q.bls.sdsu-4AL* identified in our study were in a region similar to the QTLs reported by Adhikari et al. (2012b) in the GWAS analysis for BLS resistance in spring wheat*,* whereas another QTL, *Q.bls.sdsu-1BS* in a similar region as reported by Kandel et al. (2015). Our study not only validated these three QTLs (*Q.bls.sdsu-1AL*, *Q.bls.sdsu-1BS*, and *Q.bls.sdsu-4AL*) but with higher marker coverage, we also identified SNPs associated with these QTLs. Due to low marker density and SSR or DArT markers used in these studies, we could not perform a precise comparison. Two novel QTLs, *Q.bls.sdsu-3AL* and *Q.bls.sdsu-7AS* identified in this study have not been reported previously. However, genomic regions conferring resistance to BLS reported on chromosomes 4B and 6B (Adhikari et al., 2012b) and chromosomes 2A and 6B (Kandel et al., 2015) were not significant in our HWWAMP. Two QTLs (*Q.bls.sdsu-3AL,* and *Q.bls.sdsu-4AL*) of the five QTLs we identified were mapped in syntenic regions when compared to rice, that further validates the role of these two QTLs in BLS resistance and suggests comparative genomics could also help in further fine mapping of these QTLs and understanding of the mechanism of BLS resistance in cereals.

The production of PR proteins in response to pathogens are the primary mechanisms in inducing plant’s self-defense system (Ebrahim, Usha & Singh, 2011). Several PR proteins have been characterized in recent years, and in general, they have been classified into at least 17 protein families. However, there are several pathogenesis-related proteins that do not constitute a superfamily of proteins (Dangl & Jones, 2001; Sels et al., 2008). Our functional annotation analysis of the candidate regions identified several genes which code for LRR, NB-ARC, Pkinase, and Pkinase_Tyr type proteins. Several bacterial resistance genes have been cloned and characterized in other plant species like *Oryza sativa* (Song et al., 1995; Yoshimura et al., 1998; Iyer & McCouch, 2004; Bimolata et al., 2013; Tian et al., 2014), *Zea mays* (Zhao et al., 2005), and *Solanum lycopersicum* (Martin et al., 1994; Zhou et al., 1995; Salmeron et al., 1996; Schornack et al., 2004). Bacterial blight resistance genes *Xa21* and *Xa26* in rice consist of an extracellular leucine-rich repeat (LRR) domain and a cytoplasmic receptor-like kinase domain (Song et al., 1995; Sun, Cao & Wang, 2006). The NBS is the most common R-gene known to confer resistance against bacterial pathogens, it has a highly conserved NBS domain that can bind and hydrolyze ATP or GTP (Wan et al., 2012; Zhang et al., 2016). We also identified several NB-ARC type proteins in candidate regions (Figs. 3D and 4D) which is a larger entity that includes nucleotide-binding fold in NB-LRR proteins and known to play important role in plant defense (DeYoung & Innes, 2006; Malosetti et al., 2007; Van Ooijen et al., 2008). In addition, many protein kinase coding genes were identified in all candidate regions. Several Pkinase genes have been known to play a role in bacterial resistance like *OsMPK6* in rice (Ueno et al., 2013) and *Pto* in tomato (Martin et al., 1994).

The goal of molecular characterization of BLS resistance was to identify genes or QTLs conferring BLS resistance and our findings will help in further understanding the underlying molecular network and their interactions to achieve resistance responses. The SNPs linked to the five BLS resistance QTLs identified in our study (Table 1) will facilitate in the selection of resistant individuals for backcrossing or pyramiding of favorable alleles into high yielding adapted winter wheat cultivars. The sequence flanking these SNPs is provided in Table S6 and can be used to design KBioscience Competitive Allele-Specific Polymerase chain reaction (KASPar) based markers and help in the development of BLS resistant wheat cultivars.

## Conclusions

In the current study, we identified sources of resistance to Bacterial Leaf Streak in hard winter wheat germplasm and characterized five genomic regions associated with resistance to BLS in HWWAMP. Further, the SNP markers associated with these genomic regions would be useful in marker-assisted selection to develop BLS resistant wheat varieties. The information from this work will help enhance our understanding of the molecular basis of BLS resistance in wheat.

##  Supplemental Information

10.7717/peerj.7276/supp-1 Figure S1 The geographic diversity of 299 accessions of the hard winter wheat association mapping panel (HWWAMP). Note: Besides 298 genotypes from the USA, one genotype is from Ukraine

10.7717/peerj.7276/supp-2 Figure S2 Diagrammatic representation of the categorization used to evaluate the lines in the Hard Winter Wheat Association Mapping Panel (HWWAMP) against *X. campestris pv. translucens* The dotted lines represent the bacterial increase from the infiltrated region while the solid lines represent the initial region of infiltration.

10.7717/peerj.7276/supp-3 Figure S3 Distribution of 15,590 SNP markers across the 21 wheat chromosomes. Chromosomal locations and positions of SNP markers are obtained from the wheat 90K consensus genetic map

10.7717/peerj.7276/supp-4 Figure S4 Synteny analysis of wheat chromosomes 3A and 4A against corresponding rice chromosomes R1 and R3 along with the location of their respective bacterial leaf streak (BLS) resistance QTLs

10.7717/peerj.7276/supp-5 Table S1 The reaction of 299 accessions of the hard winter wheat association mapping panel (HWWAMP) to bacterial leaf streak (BLS) in the two greenhouse (two replications) and field (two replications) experiments

10.7717/peerj.7276/supp-6 Table S2 Analysis of variance of bacterial leaf streak (BLS) score for 299 hard winter wheat association mapping panel (HWWAMP) genotypes grown in two greenhouse experiments

10.7717/peerj.7276/supp-7 Table S3 Analysis of variance of bacterial leaf streak (BLS) score for 299 hard winter wheat association mapping panel (HWWAMP) genotypes in the two replications conducted in the field experiment

10.7717/peerj.7276/supp-8 Table S4 Summary of significant SNPs linked to QTLs for bacterial leaf streak (BLS) resistance detected from the best linear unbiased estimator (BLUE) values, Greenhouse (GH), and Field scores (Field)

10.7717/peerj.7276/supp-9 Table S5 Plant disease resistance related genes predicted in the five genomic regions that span the bacterial leaf streak (BLS) resistance QTLs *Q.bls.sdsu-1AL*,* Q.bls.sdsu-1BS*, *Q.bls.sdsu-3AL*, *Q.bls.sdsu-4AL*, and *Q.bls.sdsu-7AS*

10.7717/peerj.7276/supp-10 Table S6 Nucleotide sequence flanking the SNPs associated with bacterial leaf streak (BLS) resistance in the hard winter wheat association mapping panel (HWWAMP)

10.7717/peerj.7276/supp-11 Table S7 The raw data for reaction of 299 accessions of the hard winter wheat association mapping panel (HWWAMP) to bacterial leaf streak (BLS) in the two greenhouse experiments (two replications each) and field experiment (two replications)

10.7717/peerj.7276/supp-12 Table S8 Additional information about 299 Hard winter wheat association mapping panel (HWWAMP) lines

## Additional Information and Declarations

### DNA Deposition

The following information was supplied regarding the deposition of DNA sequences: The SNP data is available at https://triticeaetoolbox.org/wheat/genotyping/display_genotype.php?trial_code=TCAP90K_HWWAMP.

### Data Availability

The following information was supplied regarding data availability: The raw BLS data for each experiment are available in Table S7.
