# Supplementary figures and images for: Molecular characterization of bacterial leaf streak resistance in hard winter wheat
Source: PeerJ. 2019 Jul 15;7:e7276. doi: 10.7717/peerj.7276 (PMC6637926; doi:10.7717/peerj.7276)

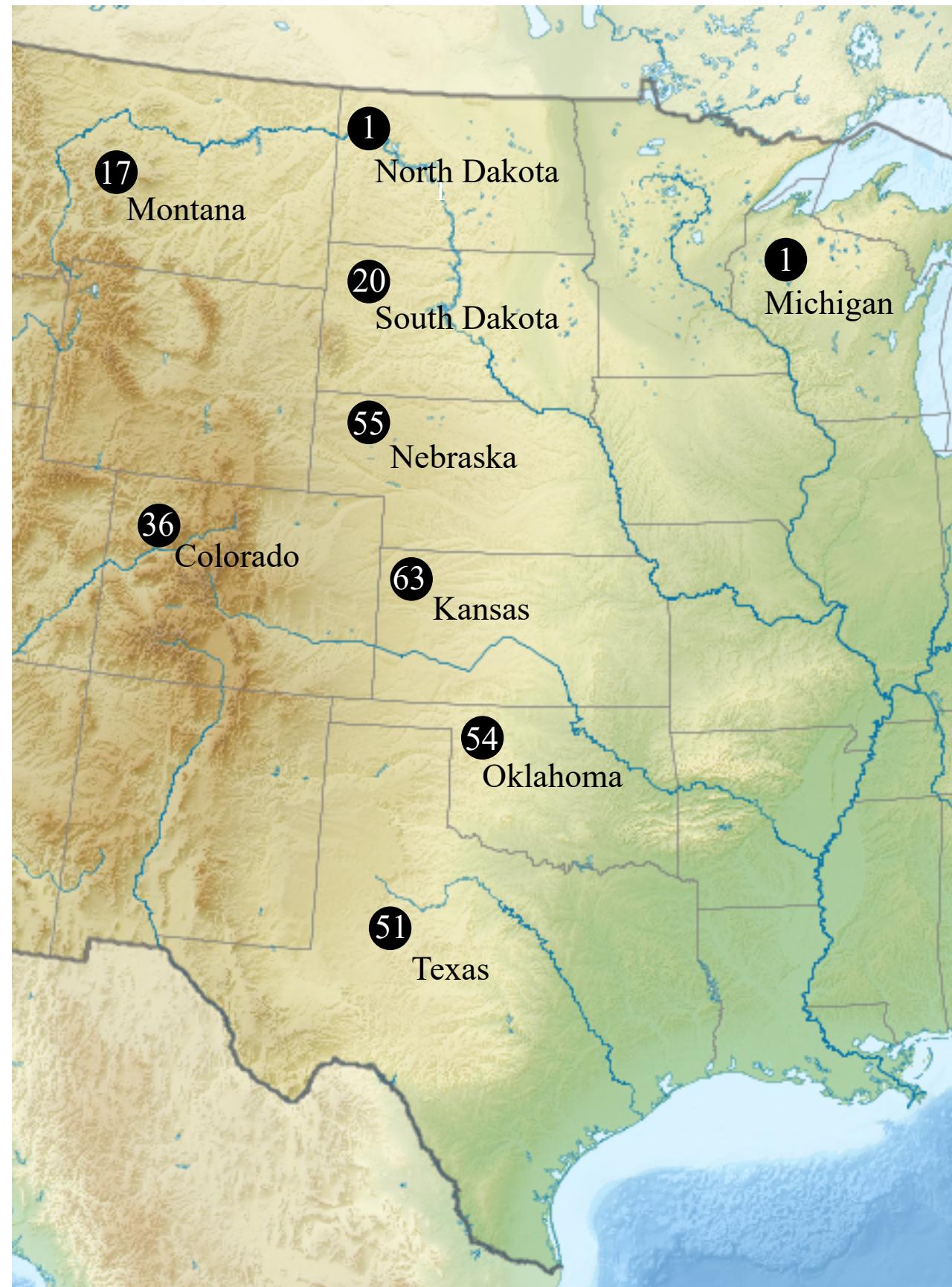

Supplement: Figure S1 [file peerj-07-7276-s001.pdf]

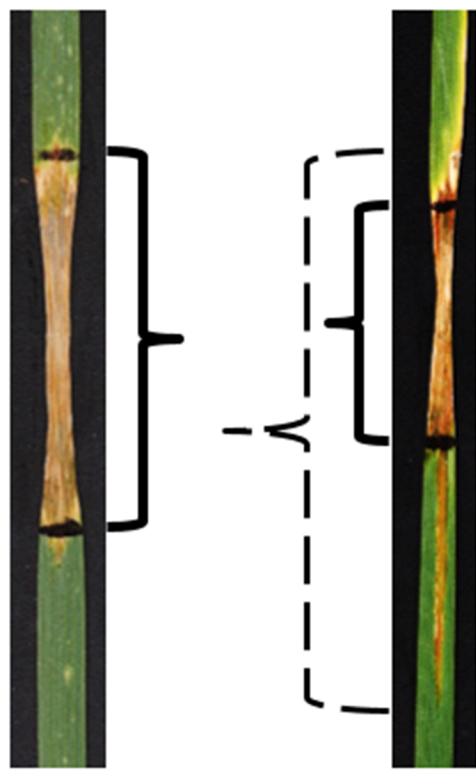

R

S

**Distance increase** = Bacterial  
increase (dotted line) – initial  
infiltrated region (solid line)

Supplement: Figure S2 — The dotted lines represent the bacterial increase from the infiltrated region while the solid lines represent the initial region of infiltration. [file peerj-07-7276-s002.pdf]

SNP Markers

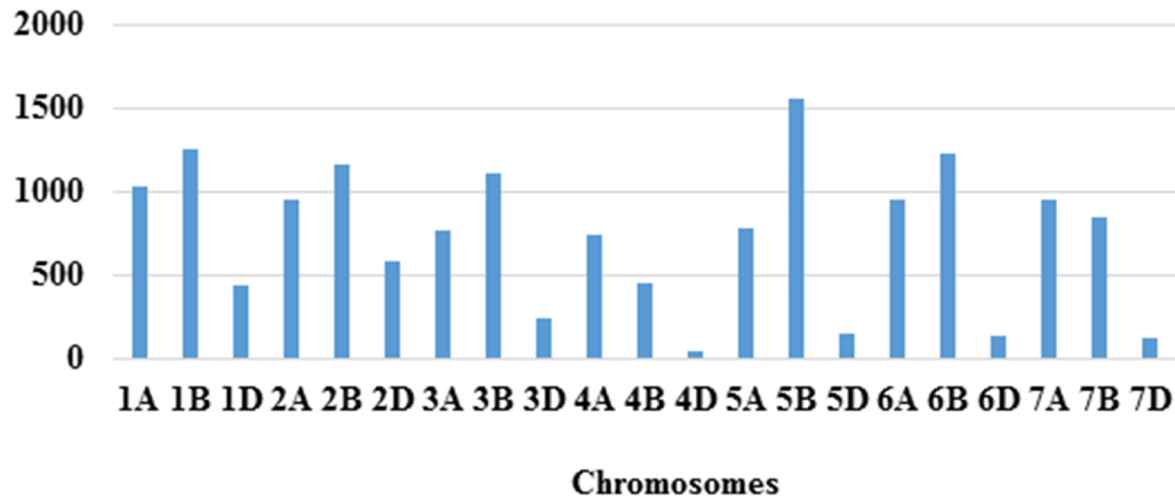

Supplement: Figure S3 [file peerj-07-7276-s003.pdf]

Rice

Wheat

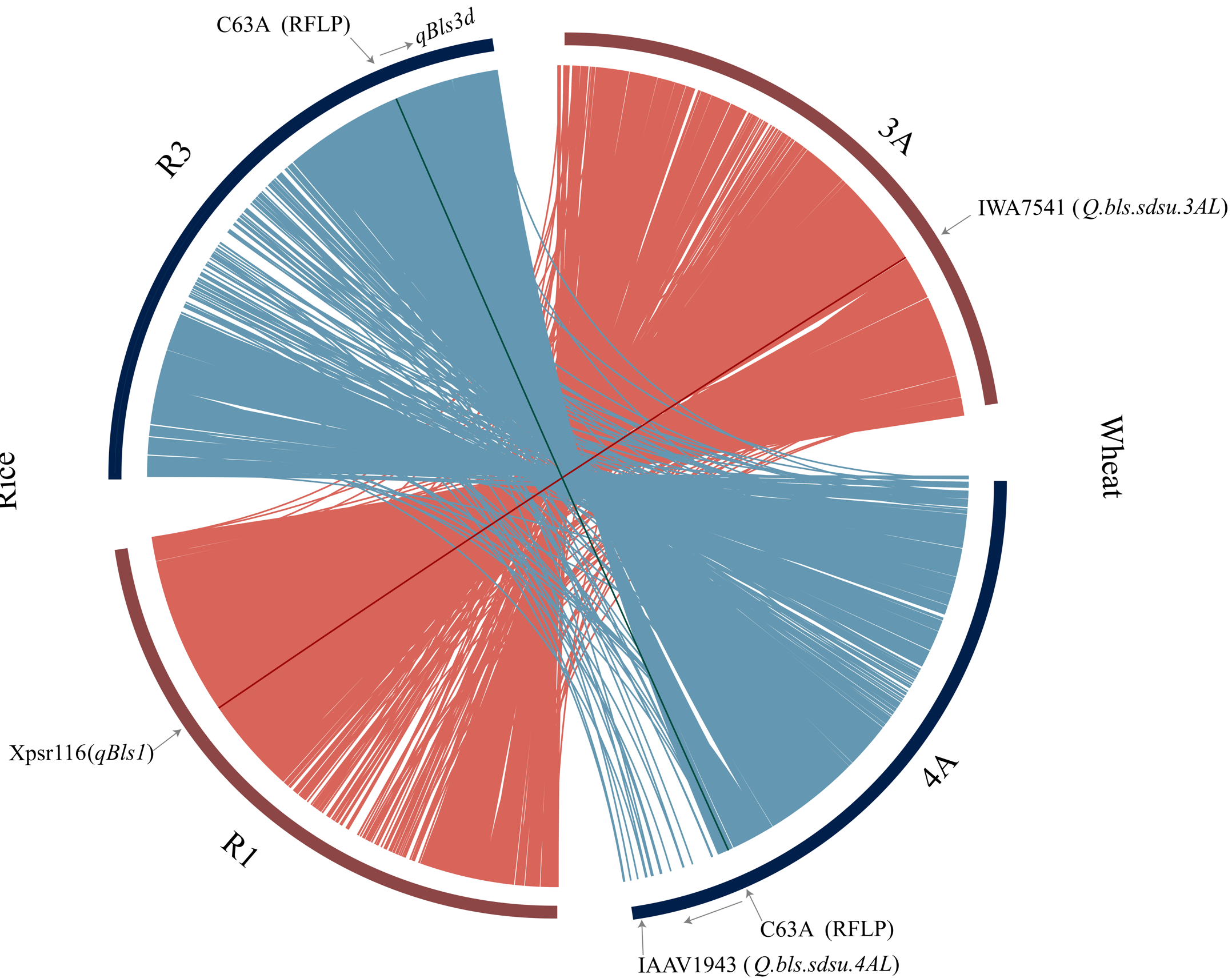

Supplement: Figure S4 [file peerj-07-7276-s004.pdf]
