# Supplementary material for: Molecular characterization of bacterial leaf streak resistance in hard winter wheat
Source: PeerJ. 2019 Jul 15;7:e7276. doi: 10.7717/peerj.7276 (PMC6637926; doi:10.7717/peerj.7276)
Supplement: Table S1 [file peerj-07-7276-s005.docx]

Supplementary Table 1. The reaction of 299 accessions of the hard winter wheat association mapping panel (HWWAMP) to bacterial leaf streak (BLS) in the two greenhouse (two replications) and field (two replications) experiments.

| **Genotype** | **ID** | **GH Experiment 1**  **Mean score** | **GH Experiment 2**  **Mean score** | **Field**  **Mean score** | **BLUEs** |
| --- | --- | --- | --- | --- | --- |
| 1 | TRIUMPH64 | 3 | 3 | 3 | 3 |
| 2 | CHISHOLM | 2 | 2 | 2 | 2 |
| 3 | CENTURY | 3 | 3 | 3 | 3 |
| 4 | CUSTER | 3 | 3 | 3 | 3 |
| 5 | 2174-05 | 3 | 3 | 3 | 3 |
| 6 | INTRADA | 3 | 3 | 3 | 3 |
| 7 | OK101 | 3 | 3 | 3 | 3 |
| 8 | OK102 | 2 | 2 | 2 | 2 |
| 9 | ENDURANCE | 3 | 3 | 3 | 3 |
| 10 | DELIVER | 3 | 3 | 3 | 3 |
| 11 | OK_BULLET | 2 | 2 | 2 | 2 |
| 12 | CENTERFIELD | 2 | 2 | 2 | 2 |
| 13 | GUYMON | 3 | 3 | 3 | 3 |
| 14 | DUSTER | 3 | 3 | 3 | 3 |
| 15 | OK_RISING | 3 | 3 | 3 | 3 |
| 16 | OK02405 | 3 | 3 | 3 | 3 |
| 17 | PETE | 3 | 3 | 3 | 3 |
| 18 | BILLINGS | 3 | 3 | 3 | 3 |
| 19 | OK04505 | 2 | 2 | 2 | 2 |
| 20 | OK04525 | 2 | 2 | 2 | 2 |
| 21 | OK04507 | 3 | 3 | 3 | 3 |
| 22 | OK05830 | 3 | 3 | 3 | 3 |
| 23 | OK04111 | 3 | 3 | 3 | 3 |
| 24 | OK04415 | 2 | 2 | 2 | 2 |
| 25 | OK05711W | 3 | 3 | 3 | 3 |
| 26 | OK05723W | 1 | 1 | 1 | 1 |
| 27 | OK05108 | 4 | 4 | 5 | 5 |
| 28 | OK05122 | 3 | 3 | 3 | 3 |
| 29 | OK05526 | 4 | 4 | 5 | 5 |
| 30 | OK05134 | 2 | 2 | 2 | 2 |
| 31 | OK05303 | 3 | 3 | 3 | 3 |
| 32 | OK05312 | 3 | 3 | 3 | 3 |
| 33 | OK05511 | 2 | 2 | 2 | 2 |
| 34 | OK05204 | 2 | 2 | 2 | 2 |
| 35 | GARRISON | 2 | 2 | 2 | 2 |
| 36 | OK06114 | 2 | 2 | 2 | 2 |
| 37 | OK06210 | 2 | 2 | 2 | 2 |
| 38 | OK06319 | 4 | 4 | 5 | 5 |
| 39 | OK06318 | 3 | 3 | 3 | 3 |
| 40 | OK06336 | 3 | 3 | 3 | 3 |
| 41 | AGATE | 3 | 3 | 3 | 3 |
| 42 | ALLIANCE | 3 | 3 | 3 | 3 |
| 43 | ANTELOPE | 5 | 5 | 5 | 5 |
| 44 | ARAPAHOE | 3 | 3 | 3 | 3 |
| 45 | BENNETT | 3 | 3 | 3 | 3 |
| 46 | BUCKSKIN | 2 | 2 | 2 | 2 |
| 47 | CENTURA | 2 | 2 | 2 | 2 |
| 48 | CENTURK78 | 3 | 3 | 3 | 3 |
| 49 | CHEYENNE | 3 | 3 | 3 | 3 |
| 50 | COLT | 2 | 2 | 2 | 2 |
| 51 | COUGAR | 2 | 2 | 2 | 2 |
| 52 | CULVER | 2 | 2 | 2 | 2 |
| 53 | GAGE | 2 | 2 | 2 | 2 |
| 54 | GOODSTREAK | 1 | 1 | 1 | 1 |
| 55 | HALLAM | 2 | 2 | 2 | 2 |
| 56 | HARRY | 2 | 2 | 2 | 2 |
| 57 | HOMESTEAD | 2 | 2 | 3 | 2.5 |
| 58 | INFINITY_CL | 2 | 2 | 3 | 2.5 |
| 59 | KHARKOF | 3 | 3 | 3 | 3 |
| 60 | MILLENNIUM | 2 | 2 | 3 | 3 |
| 61 | CAMELOT | 3 | 3 | 3 | 3 |
| 62 | OVERLAND | 2 | 3 | 3 | 3 |
| 63 | NE99495 | 3 | 3 | 3 | 3 |
| 64 | NIOBRARA | 2 | 3 | 3 | 3 |
| 65 | NUPLAINS | 3 | 3 | 3 | 3 |
| 66 | PRONGHORN | 3 | 3 | 3 | 3 |
| 67 | RAWHIDE | 2 | 3 | 3 | 3 |
| 68 | REDLAND | 3 | 3 | 3 | 3 |
| 69 | SCOUT66 | 1 | 1 | 1 | 1 |
| 70 | SIOUXLAND | 3 | 4 | 4 | 4 |
| 71 | TURKEY_NEBSEL | 2 | 3 | 3 | 3 |
| 72 | VISTA | 1 | 1 | 1 | 1 |
| 73 | WAHOO | 2 | 3 | 3 | 3 |
| 74 | WARRIOR | 2 | 3 | 3 | 3 |
| 75 | WESLEY | 3 | 4 | 4 | 4 |
| 76 | WICHITA | 3 | 4 | 4 | 3.5 |
| 77 | WINDSTAR | 3 | 4 | 4 | 4 |
| 78 | JAGGER | 3 | 4 | 4 | 4 |
| 79 | LANCER | 2 | 3 | 3 | 3 |
| 80 | SETTLER_CL | 2 | 3 | 3 | 3 |
| 81 | ANTON | 4 | 4 | 4 | 4 |
| 82 | MACE | 4 | 4 | 4 | 4 |
| 83 | JERRY | 4 | 4 | 4 | 4 |
| 84 | TAM107-R7 | 5 | 5 | 5 | 5 |
| 85 | ARLIN | 4 | 4 | 4 | 4 |
| 86 | ALICE | 2 | 3 | 3 | 3 |
| 87 | DARRELL | 2 | 3 | 3 | 3 |
| 88 | EXPEDITION | 2 | 3 | 3 | 3 |
| 89 | WENDY | 1 | 1 | 1 | 1 |
| 90 | SD00111-9 | 2 | 3 | 3 | 3 |
| 91 | SD01237 | 3 | 3 | 3 | 3 |
| 92 | SD01058 | 4 | 4 | 4 | 4 |
| 93 | SD05118 | 4 | 4 | 4 | 4 |
| 94 | SD05210 | 3 | 3 | 3 | 3 |
| 95 | SD05W018 | 3 | 3 | 3 | 3 |
| 96 | NEKOTA | 3 | 3 | 3 | 3 |
| 97 | TANDEM | 4 | 4 | 4 | 4 |
| 98 | CRIMSON | 4 | 4 | 4 | 4 |
| 99 | ROSE | 4 | 4 | 4 | 4 |
| 100 | DAWN | 3 | 3 | 3 | 3 |
| 101 | WINOKA | 3 | 3 | 3 | 3 |
| 102 | NELL | 5 | 5 | 5 | 5 |
| 103 | RITA | 4 | 4 | 4 | 4 |
| 104 | BRONZE | 3 | 3 | 3 | 3 |
| 105 | HUME | 3 | 3 | 3 | 3 |
| 106 | GENT | 4 | 4 | 4 | 4 |
| 107 | HARDING | 3 | 3 | 3 | 3 |
| 108 | HV9W03-1551WP | 4 | 4 | 4 | 4 |
| 109 | G1878 | 4 | 4 | 4 | 4 |
| 110 | HV9W03-1379R | 3 | 3 | 3 | 3 |
| 111 | HV9W03-1596R | 4 | 4 | 4 | 4 |
| 112 | HV9W05-1280R | 4 | 4 | 4 | 4 |
| 113 | HV9W06-504 | 4 | 4 | 4 | 4 |
| 114 | SPARTAN | 4 | 4 | 4 | 4 |
| 115 | HV906-865 | 3 | 3 | 3 | 3 |
| 116 | TARKIO | 4 | 4 | 4 | 4 |
| 117 | SMOKYHILL | 4 | 4 | 4 | 4 |
| 118 | SHOCKER | 4 | 4 | 4 | 4 |
| 119 | VONA | 3 | 3 | 3 | 3 |
| 120 | CO940610 | 3 | 3 | 3 | 3 |
| 121 | AVALANCHE | 4 | 4 | 4 | 4 |
| 122 | BOND_CL | 4 | 4 | 4 | 4 |
| 123 | PLATTE | 3 | 3 | 3 | 3 |
| 124 | LINDON | 4 | 4 | 4 | 4 |
| 125 | CO03W043 | 3 | 3 | 3 | 3 |
| 126 | CO03W054 | 4 | 4 | 4 | 4 |
| 127 | THUNDER_CL | 3 | 3 | 3 | 3 |
| 128 | CO04025 | 4 | 4 | 4 | 4 |
| 129 | CO04393 | 3 | 3 | 3 | 3 |
| 130 | CO04499 | 4 | 4 | 4 | 4 |
| 131 | CO04W320 | 4 | 4 | 4 | 4 |
| 132 | LAMAR | 3 | 3 | 3 | 3 |
| 133 | CARSON | 4 | 4 | 4 | 4 |
| 134 | HAIL | 4 | 4 | 4 | 4 |
| 135 | SANDY | 3 | 3 | 3 | 3 |
| 136 | DUKE | 4 | 4 | 4 | 4 |
| 137 | HALT | 3 | 3 | 3 | 3 |
| 138 | HATCHER | 3 | 3 | 3 | 3 |
| 139 | PRAIRIE_RED | 3 | 3 | 3 | 3 |
| 140 | YUMAR | 4 | 4 | 4 | 4 |
| 141 | ABOVE | 3 | 3 | 3 | 3 |
| 142 | CO03064 | 3 | 3 | 3 | 3 |
| 143 | BILL_BROWN | 3 | 3 | 3 | 3 |
| 144 | RIPPER | 4 | 4 | 4 | 4 |
| 145 | PROWERS | 3 | 3 | 3 | 3 |
| 146 | AKRON | 4 | 4 | 4 | 4 |
| 147 | JULES | 4 | 4 | 4 | 4 |
| 148 | YUMA | 4 | 4 | 4 | 4 |
| 149 | TAMW-101 | 4 | 4 | 4 | 4 |
| 150 | TAM105 | 4 | 4 | 4 | 4 |
| 151 | TAM107 | 3 | 3 | 3 | 3 |
| 152 | TAM109 | 4 | 4 | 4 | 4 |
| 153 | TAM110 | 4 | 4 | 4 | 4 |
| 154 | TAM111 | 4 | 4 | 4 | 4 |
| 155 | TAM112 | 3 | 3 | 3 | 3 |
| 156 | TAM200 | 4 | 4 | 4 | 4 |
| 157 | TAM202 | 3 | 3 | 3 | 3 |
| 158 | TAM203 | 4 | 4 | 4 | 4 |
| 159 | TAM302 | 3 | 3 | 3 | 3 |
| 160 | TAM303 | 4 | 4 | 4 | 4 |
| 161 | TAM304 | 5 | 5 | 5 | 5 |
| 162 | TAM400 | 3 | 3 | 3 | 3 |
| 163 | LOCKETT | 5 | 5 | 5 | 5 |
| 164 | STURDY | 3 | 3 | 3 | 3 |
| 165 | STURDY_2K | 4 | 4 | 4 | 4 |
| 166 | MIT | 5 | 5 | 5 | 5 |
| 167 | CAPROCK | 3 | 3 | 3 | 3 |
| 168 | TX01A5936 | 4 | 4 | 4 | 4 |
| 169 | TAM401 | 3 | 3 | 3 | 3 |
| 170 | TX02A0252 | 3 | 3 | 3 | 3 |
| 171 | TX03A0148 | 3 | 3 | 3 | 3 |
| 172 | TX03A0563 | 3 | 3 | 3 | 3 |
| 173 | TX04A001246 | 3 | 3 | 3 | 3 |
| 174 | TX01V5134RC-3 | 4 | 4 | 4 | 4 |
| 175 | TX04M410164 | 4 | 4 | 4 | 4 |
| 176 | TX04M410211 | 3 | 3 | 3 | 3 |
| 177 | TX04V075080 | 4 | 4 | 4 | 4 |
| 178 | TX99A0153-1 | 3 | 3 | 3 | 3 |
| 179 | TX01M5009-28 | 3 | 3 | 3 | 3 |
| 180 | TX00V1131 | 3 | 3 | 3 | 3 |
| 181 | TX99U8618 | 3 | 3 | 3 | 3 |
| 182 | TX96D1073 | 4 | 4 | 4 | 4 |
| 183 | 2180 | 4 | 4 | 4 | 4 |
| 184 | HG-9 | 4 | 4 | 4 | 4 |
| 185 | TX86A5606 | 4 | 4 | 4 | 4 |
| 186 | TX86A6880 | 3 | 3 | 3 | 3 |
| 187 | TX86A8072 | 4 | 4 | 4 | 4 |
| 188 | CREST | 4 | 4 | 4 | 4 |
| 189 | ROSEBUD | 4 | 4 | 4 | 4 |
| 190 | JUDITH | 3 | 3 | 3 | 3 |
| 191 | MT85200 | 4 | 4 | 4 | 4 |
| 192 | NUSKY | 3 | 3 | 3 | 3 |
| 193 | MT9513 | 4 | 4 | 4 | 4 |
| 194 | MT9904 | 3 | 3 | 3 | 3 |
| 195 | MT9982 | 3 | 3 | 3 | 3 |
| 196 | GENOU | 4 | 4 | 4 | 4 |
| 197 | NORRIS | 4 | 4 | 4 | 4 |
| 198 | YELLOWSTONE | 3 | 3 | 3 | 3 |
| 199 | MT0495 | 4 | 4 | 4 | 4 |
| 200 | MTS0531 | 4 | 4 | 4 | 4 |
| 201 | DECADE | 3 | 3 | 3 | 3 |
| 202 | MT06103 | 4 | 4 | 4 | 4 |
| 203 | JUDEE | 3 | 3 | 3 | 3 |
| 204 | LAKIN | 3 | 3 | 3 | 3 |
| 205 | STANTON | 4 | 4 | 4 | 4 |
| 206 | TREGO | 3 | 3 | 3 | 3 |
| 207 | KARL_92 | 4 | 4 | 4 | 4 |
| 208 | DODGE | 3 | 3 | 3 | 3 |
| 209 | NORKAN | 3 | 3 | 3 | 3 |
| 210 | CHENEY | 3 | 3 | 3 | 3 |
| 211 | NEWTON | 3 | 3 | 3 | 3 |
| 212 | LARNED | 2 | 2 | 2 | 2 |
| 213 | PARKER76 | 3 | 3 | 3 | 3 |
| 214 | KIRWIN | 4 | 4 | 4 | 4 |
| 215 | SAGE | 3 | 3 | 3 | 3 |
| 216 | TRISON | 4 | 4 | 4 | 4 |
| 217 | EAGLE | 1 | 1 | 1 | 1 |
| 218 | SHAWNEE | 4 | 4 | 4 | 4 |
| 219 | PARKER | 3 | 3 | 3 | 3 |
| 220 | KAW61 | 4 | 4 | 4 | 4 |
| 221 | TASCOSA | 4 | 4 | 4 | 4 |
| 222 | BISON | 4 | 4 | 4 | 4 |
| 223 | KIOWA | 4 | 4 | 4 | 4 |
| 224 | COMANCHE | 3 | 3 | 3 | 3 |
| 225 | BAKERS_WHITE | 4 | 4 | 4 | 4 |
| 226 | BURCHETT | 3 | 3 | 3 | 3 |
| 227 | CUTTER | 3 | 3 | 3 | 3 |
| 228 | DUMAS | 4 | 4 | 4 | 4 |
| 229 | HONDO | 4 | 4 | 4 | 4 |
| 230 | JAGALENE | 3 | 3 | 3 | 3 |
| 231 | LONGHORN | 3 | 3 | 3 | 3 |
| 232 | NEOSHO | 4 | 4 | 4 | 4 |
| 233 | OGALLALA | 4 | 4 | 4 | 4 |
| 234 | POSTROCK | 3 | 3 | 3 | 3 |
| 235 | THUNDERBOLT | 3 | 3 | 3 | 3 |
| 236 | W04-417 | 4 | 4 | 4 | 4 |
| 237 | NUFRONTIER | 3 | 3 | 3 | 3 |
| 238 | NUHORIZON | 3 | 3 | 3 | 3 |
| 239 | ONAGA | 3 | 3 | 3 | 3 |
| 240 | RONL | 3 | 3 | 3 | 3 |
| 241 | 2145 | 3 | 3 | 3 | 3 |
| 242 | HEYNE | 3 | 3 | 3 | 3 |
| 243 | KS00F5-20-3 | 4 | 4 | 5 | 4.5 |
| 244 | OVERLEY | 4 | 4 | 5 | 5 |
| 245 | FULLER | 3 | 3 | 3 | 3 |
| 246 | COSSACK | 4 | 5 | 5 | 5 |
| 247 | ENHANCER | 3 | 3 | 3 | 3 |
| 248 | SANTA_FE | 4 | 5 | 5 | 5 |
| 249 | VENANGO | 4 | 5 | 5 | 5 |
| 250 | WB411W | 3 | 3 | 3 | 3 |
| 251 | KEOTA | 3 | 3 | 3 | 3 |
| 252 | TX05A001822 | 4 | 5 | 5 | 5 |
| 253 | TX06A001263 | 3 | 3 | 3 | 3 |
| 254 | TX06A001132 | 3 | 3 | 3 | 3 |
| 255 | TX06A001281 | 4 | 5 | 5 | 5 |
| 256 | TX06A001386 | 3 | 3 | 3 | 3 |
| 257 | TX05V7259 | 5 | 5 | 5 | 5 |
| 258 | TX05V7269 | 3 | 3 | 3 | 3 |
| 259 | TX05A001188 | 3 | 3 | 3 | 3 |
| 260 | TX07A001279 | 5 | 5 | 5 | 5 |
| 261 | TX07A001318 | 3 | 3 | 3 | 3 |
| 262 | TX07A001420 | 1 | 1 | 1 | 1 |
| 263 | TX06V7266 | 3 | 3 | 3 | 3 |
| 264 | OK1067071 | 3 | 3 | 3 | 3 |
| 265 | OK1067274 | 5 | 5 | 5 | 5 |
| 266 | OK1068002 | 3 | 3 | 3 | 3 |
| 267 | OK1068009 | 5 | 5 | 5 | 5 |
| 268 | OK1068026 | 3 | 3 | 3 | 3 |
| 269 | OK1068112 | 1 | 1 | 1 | 1 |
| 270 | OK1070275 | 3 | 3 | 3 | 3 |
| 271 | OK1070267 | 5 | 5 | 5 | 5 |
| 272 | OK09634 | 5 | 5 | 5 | 5 |
| 273 | OK10119 | 5 | 5 | 5 | 5 |
| 274 | GALLAGHER | 3 | 3 | 3 | 3 |
| 275 | OK07231 | 3 | 3 | 3 | 3 |
| 276 | OK07S117 | 3 | 3 | 3 | 3 |
| 277 | OK08328 | 5 | 5 | 5 | 5 |
| 278 | BIG_SKY | 3 | 3 | 3 | 3 |
| 279 | DANBY | 3 | 3 | 3 | 3 |
| 280 | E2041 | 3 | 3 | 3 | 3 |
| 281 | DENALI | 5 | 5 | 5 | 5 |
| 282 | CO050337-2 | 3 | 3 | 3 | 3 |
| 283 | BYRD | 5 | 5 | 5 | 5 |
| 284 | CO07W245 | 3 | 3 | 3 | 3 |
| 285 | MCGILL | 3 | 3 | 3 | 3 |
| 286 | NE02558 | 3 | 3 | 3 | 3 |
| 287 | NW03666 | 5 | 5 | 5 | 5 |
| 288 | NE04490 | 1 | 1 | 1 | 1 |
| 289 | NE05430 | 3 | 3 | 3 | 3 |
| 290 | NE05496 | 3 | 3 | 3 | 3 |
| 291 | NE05548 | 5 | 5 | 5 | 5 |
| 292 | NE06545 | 5 | 5 | 5 | 5 |
| 293 | NE06607 | 5 | 5 | 5 | 5 |
| 294 | ROBIDOUX | 1 | 1 | 1 | 1 |
| 295 | NI06736 | 3 | 3 | 3 | 3 |
| 296 | NI06737 | 5 | 5 | 5 | 5 |
| 297 | NI07703 | 3 | 3 | 3 | 3 |
| 298 | NI08707 | 3 | 3 | 3 | 3 |
| 299 | NI08708 | 3 | 3 | 3 | 3 |
| Mean |  | 3.24 | 3.31 | 3.34 | 3.33 |
