# Supplementary material for: Molecular characterization of bacterial leaf streak resistance in hard winter wheat
Source: PeerJ. 2019 Jul 15;7:e7276. doi: 10.7717/peerj.7276 (PMC6637926; doi:10.7717/peerj.7276)
Supplement: Table S2 [file peerj-07-7276-s006.docx]

Supplementary Table 2. Analysis of variance of bacterial leaf streak (BLS) score for 299 hard winter wheat association mapping panel (HWWAMP) genotypes grown in two greenhouse experiments.

| Source | df | MSS | F-value | P-value |
| --- | --- | --- | --- | --- |
| Genotype | 298 | 3.01 | 78.92 | < 2.00e^-16*^ |
| Experiment | 1 | 2.00 | 52.53 | 9.15e^-13*^ |
| Genotype*Experiment | 298 | 0.03 | 0.91 | 0.8014 |
| Residuals | 896 | 0.03 |  |  |

^*^Significant at α-level of 0.05.
