# Supplementary material for: Molecular characterization of bacterial leaf streak resistance in hard winter wheat
Source: PeerJ. 2019 Jul 15;7:e7276. doi: 10.7717/peerj.7276 (PMC6637926; doi:10.7717/peerj.7276)
Supplement: Table S3 [file peerj-07-7276-s007.docx]

Supplementary Table 3. Analysis of variance of bacterial leaf streak (BLS) score for 299 hard winter wheat association mapping panel (HWWAMP) genotypes in the two replications conducted in the field experiment.

| Source | df | MSS | F-value | P-value |
| --- | --- | --- | --- | --- |
| Genotype | 298 | 1.56 | 39.17 | < 2.00e^-16*^ |
| Residuals | 299 | 0.04 |  |  |

^*^Significant at α-level of 0.05.
