# Supplementary material for: Molecular characterization of bacterial leaf streak resistance in hard winter wheat
Source: PeerJ. 2019 Jul 15;7:e7276. doi: 10.7717/peerj.7276 (PMC6637926; doi:10.7717/peerj.7276)
Supplement: Table S5 [file peerj-07-7276-s009.docx]

Supplementary Table 5. Plant disease resistance related genes predicted in the five genomic regions that span the bacterial leaf streak (BLS) resistance QTLs *Q.bls.sdsu-1AL*, *Q.bls.sdsu-1BS*, *Q.bls.sdsu-3AL*, *Q.bls.sdsu-4AL*, and *Q.bls.sdsu-7AS*.

| QTL | Gene ID | Chromosome | Location on pseudomolecule (bp) | Predicted Function/ Description |
| --- | --- | --- | --- | --- |
| *Q.bls.sdsu-1AL* | TraesCS1A02G422600 | 1AL | 578014400-57801764 | RL_P_Kinase |
|  | TraesCS1A02G422700 | 1AL | 578021877-57802503 | RL_P_Kinase |
|  | TraesCS1A02G422800 | 1AL | 578196408-57819939 | RL_P_Kinase |
|  | TraesCS1A02G423400 | 1AL | 578812352-57881415 | MAP_Kinase |
|  | TraesCS1A02G423500 | 1AL | 578830962-57883129 | TIR-NBS-LRR |
|  | TraesCS1A02G424900 | 1AL | 579861533-57986360 | P_Kinase |
|  | TraesCS1A02G425800 | 1AL | 580571415-58057268 | NB-ARC |
|  | TraesCS1A02G425900 | 1AL | 580660850-58066510 | NB-ARC |
|  | TraesCS1A02G426000 | 1AL | 580739950-58074366 | NB-ARC |
|  | TraesCS1A02G427300 | 1AL | 581754085-58175727 | LRR_P_Kinase |
| *Q.bls.sdsu-1BS* | TraesCS1B02G014500 | 1BS | 7040245-7047567 | NB-ARC |
|  | TraesCS1B02G014800 | 1BS | 7252011-7257065 | NB-ARC |
|  | TraesCS1B02G014900 | 1BS | 7290064-7291428 | NB-ARC |
|  | TraesCS1B02G015000 | 1BS | 7293786-7294443 | LRR |
|  | TraesCS1B02G015300 | 1BS | 7337194-7338438 | NB-ARC |
|  | TraesCS1B02G016000 | 1BS | 7632619-7638865 | NB-ARC |
|  | TraesCS1B02G016100 | 1BS | 7714907-7715746 | NB-ARC |
|  | TraesCS1B02G017100 | 1BS | 8222272-8224224 | P_Kinase |
|  | TraesCS1B02G017300 | 1BS | 8255692-8257056 | LRR |
|  | TraesCS1B02G017500 | 1BS | 8411343-8414060 | NB-ARC |
|  | TraesCS1B02G017600 | 1BS | 8556295-8560477 | NB-ARC |
|  | TraesCS1B02G018300 | 1BS | 8817404-8819921 | P_Kinase |
|  | TraesCS1B02G018500 | 1BS | 8853375-8854210 | P_Kinase |
|  | TraesCS1B02G018600 | 1BS | 8864601-8867261 | P_Kinase |
|  | TraesCS1B02G019000 | 1BS | 8990898-9007234 | NB-ARC |
|  | TraesCS1B02G019500 | 1BS | 9161801-9164311 | P_Kinase |
|  | TraesCS1B02G020300 | 1BS | 9546914-9551729 | NB-ARC |
|  | TraesCS1B02G020400 | 1BS | 9554904-9571043 | NB-ARC |
|  | TraesCS1B02G020500 | 1BS | 9578944-9581744 | WAK |
|  | TraesCS1B02G020600 | 1BS | 9592538-9599744 | RL_P_Kinase |
|  | TraesCS1B02G020700 | 1BS | 9592944-9596685 | RL_P_Kinase |
|  | TraesCS1B02G020900 | 1BS | 9691698-9692957 | NB-ARC |
|  | TraesCS1B02G021500 | 1BS | 9873851-9876007 | NB-ARC |
|  | TraesCS1B02G021600 | 1BS | 9893917-9899387 | WAK |
|  | TraesCS1B02G021800 | 1BS | 9939530-9942269 | WAK |
| *Q.bls.sdsu-3AL* | TraesCS3A02G267600 | 3AL | 492736569-49273684 | P_Kinase |
|  | TraesCS3A02G270500 | 3AL | 497654221-49766198 | LRR_P_Kinase |
|  | TraesCS3A02G271100 | 3AL | 498772638-49878814 | P_Kinase |
|  | TraesCS3A02G271300 | 3AL | 500029628-50003084 | LRR |
|  | TraesCS3A02G272900 | 3AL | 502097228-50210149 | P_Kinase |
|  | TraesCS3A02G275100 | 3AL | 504798413-50480377 | P_Kinase |
|  | TraesCS3A02G276200 | 3AL | 506227271-50623119 | RL_P_Kinase |
|  | TraesCS3A02G276600 | 3AL | 506665589-50667068 | RL_P_Kinase |
|  | TraesCS3A02G277900 | 3AL | 507469897-50747293 | P_Kinase |
|  | TraesCS3A02G278100 | 3AL | 507636683-50764089 | P_Kinase |
|  | TraesCS3A02G282800 | 3AL | 511074224-51107900 | P_Kinase |
|  | TraesCS3A02G282900 | 3AL | 511597445-51159880 | LRR_P_Kinase |
|  | TraesCS3A02G286500 | 3AL | 514557805-51456203 | RL_P_Kinase |
|  | TraesCS3A02G286900 | 3AL | 515280520-51528201 | NB-ARC |
|  | TraesCS3A02G287000 | 3AL | 515283046-51528465 | NB-ARC |
|  | TraesCS3A02G287200 | 3AL | 515723022-51572517 | NB-ARC |
|  | TraesCS3A02G287300 | 3AL | 515734859-51573550 | Fusarium resistance protein I2C5 like |
|  | TraesCS3A02G290000 | 3AL | 518905714-51890734 | LRR_P_Kinase |
|  | TraesCS3A02G290300 | 3AL | 519244293-51924790 | RL_P_Kinase |
|  | TraesCS3A02G295600 | 3AL | 529776872-52977759 | LRR |
|  | TraesCS3A02G296800 | 3AL | 531453215-53145616 | LRR |
|  | TraesCS3A02G297800 | 3AL | 532452789-53245401 | RL_P_Kinase |
| *Q.bls.sdsu-4AL* | TraesCS4A02G458900 | 4AL | 724482865-72448515 | P_Kinase |
|  | TraesCS4A02G459500 | 4AL | 725162676-72516540 | NB-ARC |
|  | TraesCS4A02G461000 | 4AL | 725910621-72591138 | LRR |
|  | TraesCS4A02G461700 | 4AL | 726212990-72621699 | NB-ARC |
|  | TraesCS4A02G461800 | 4AL | 726292466-72630015 | NB-ARC |
|  | TraesCS4A02G462800 | 4AL | 726717983-72672044 | NB-ARC |
|  | TraesCS4A02G462900 | 4AL | 726962094-72696395 | NB-ARC |
|  | TraesCS4A02G463000 | 4AL | 726963988-72696659 | NB-ARC |
| *Q.bls.sdsu-7AS* | TraesCS7A02G026400 | 7AS | 10619635-10626123 | NBS-LRR |
|  | TraesCS7A02G026900 | 7AS | 10849564-10856201 | NB-ARC |
|  | TraesCS7A02G027000 | 7AS | 10855484-10862014 | NBS-LRR |
|  | TraesCS7A02G027700 | 7AS | 11084872-11088792 | NB-ARC |
|  | TraesCS7A02G027800 | 7AS | 11091491-11101081 | NB-ARC |
|  | TraesCS7A02G028300 | 7AS | 11414317-11416000 | LRR_P_Kinase |
|  | TraesCS7A02G028400 | 7AS | 11432768-11435195 | P_Kinase |
|  | TraesCS7A02G029700 | 7AS | 12115497-12118204 | MAP_Kinase |
|  | TraesCS7A02G031300 | 7AS | 13090803-13094176 | NB-ARC |
