# Supplementary material for: Molecular characterization of bacterial leaf streak resistance in hard winter wheat
Source: PeerJ. 2019 Jul 15;7:e7276. doi: 10.7717/peerj.7276 (PMC6637926; doi:10.7717/peerj.7276)
Supplement: Table S6 [file peerj-07-7276-s010.docx]

Supplementary Table 6. Nucleotide sequence flanking the SNPs associated with bacterial leaf streak (BLS) resistance in the hard winter wheat association mapping panel (HWWAMP).

| **SNP Marker** | **QTL** | **Nucleotide Sequence** |
| --- | --- | --- |
| BS00084995_51 |  | TGACAACAACTTTGGAGTTCAAGGCGTTAATAAGAGATACGACAACTGCA**[G/T]**CAAGGAAGGTTATAACAATGCGTTTGGATTCGATAGCATAACTAAGACAT |
| Ku_c17846_363 |  | CTGGTCACTTCTTAATAGCGTCACCCGGTTGTACCTGGCATTCTCCAATA**[C/T]**TGATGAGGGAACCTTTTCATGTCTGTGTGTGTTACGTGCTCTAAGCACCC |
| IWA7541 |  | TCAAATTCATCACTGGCTAAAGCCATGCTAATTGCTTCATCCATTGAGCTTTTGTAATCATCGGTTAGCACACTGTCAAGTCCCAGATCATTTGTTTCAG**[A/G]**ATTAAGATGATTATCACCTACAGATTGCAGAATAGAAAGCACATCCTCCAAATAGAAAGTTTTGACCGGATAGGTATGGCCTGGGACTCGAATAACCGGG |
| IAAV1943 |  | ATTGACAGAGAGGAAGCGAAGCTAAAGAATGGTGGAGCGTGAGACAGATGATGGTGATGAACAATTGATTAGACCACTGCAAATTTACCCATTCTTATTA**[C/T]**TTATAAGGGAGTCTACATCCTGAAATTATGGTAGGACAGAGCTCATCTAGGTATTT |
| tplb0032m13_1358 |  | CTGCTTATGAACCCTCTTAACATCCCCAGCTCCGGGCGCCATTTCTACCT**[C/T]**GCCGTTGACCGCCTCCAGTTCAAGATGAGGACACTACTGGAGCTCCTAGG |
