# Supplementary material for: Molecular characterization of bacterial leaf streak resistance in hard winter wheat
Source: PeerJ. 2019 Jul 15;7:e7276. doi: 10.7717/peerj.7276 (PMC6637926; doi:10.7717/peerj.7276)
Supplement: Table S7 [file peerj-07-7276-s011.docx]

Supplementary Table 7. The raw data for reaction of 299 accessions of the hard winter wheat association mapping panel (HWWAMP) to bacterial leaf streak (BLS) in the two greenhouse experiments (two replications each) and field experiment (two replications).

| **Line information** | | **Greenhouse experiments** | | | | | | **Field experiment** | |
| --- | --- | --- | --- | --- | --- | --- | --- | --- | --- |
|  |  | **Experiment 1** | | **Experiment 2** | | | |  |  |
| **Sr. no.** | **Genotype** | **Rep 1** | **Rep 2** | **Rep 1** | | **Rep 2** | **Rep 1** | | **Rep 2** |
| **1** | **TRIUMPH64** | 3 | 3 | 3 | 3 | | | 3 | 3 |
| **2** | **CHISHOLM** | 2 | 2 | 2 | 2 | | | 2 | 2 |
| **3** | **CENTURY** | 3 | 3 | 3 | 3 | | | 3 | 3 |
| **4** | **CUSTER** | 3 | 3 | 3 | 3 | | | 3 | 3 |
| **5** | **2174-05** | 3 | 3 | 3 | 3 | | | 3 | 3 |
| **6** | **INTRADA** | 3 | 3 | 3 | 3 | | | 3 | 3 |
| **7** | **OK101** | 3 | 3 | 3 | 3 | | | 3 | 3 |
| **8** | **OK102** | 2 | 2 | 2 | 2 | | | 2 | 2 |
| **9** | **ENDURANCE** | 3 | 3 | 3 | 3 | | | 3 | 3 |
| **10** | **DELIVER** | 3 | 3 | 3 | 3 | | | 3 | 3 |
| **11** | **OK_BULLET** | 2 | 2 | 2 | 2 | | | 2 | 2 |
| **12** | **CENTERFIELD** | 2 | 2 | 2 | 2 | | | 2 | 2 |
| **13** | **GUYMON** | 3 | 3 | 3 | 3 | | | 3 | 3 |
| **14** | **DUSTER** | 3 | 3 | 3 | 3 | | | 3 | 3 |
| **15** | **OK_RISING** | 3 | 3 | 3 | 3 | | | 3 | 3 |
| **16** | **OK02405** | 3 | 3 | 3 | 3 | | | 3 | 3 |
| **17** | **PETE** | 3 | 3 | 3 | 3 | | | 3 | 3 |
| **18** | **BILLINGS** | 3 | 3 | 3 | 3 | | | 3 | 3 |
| **19** | **OK04505** | 2 | 2 | 2 | 2 | | | 2 | 2 |
| **20** | **OK04525** | 2 | 2 | 2 | 2 | | | 2 | 2 |
| **21** | **OK04507** | 3 | 3 | 3 | 3 | | | 3 | 3 |
| **22** | **OK05830** | 3 | 3 | 3 | 3 | | | 3 | 3 |
| **23** | **OK04111** | 3 | 3 | 3 | 3 | | | 3 | 3 |
| **24** | **OK04415** | 2 | 2 | 2 | 2 | | | 2 | 2 |
| **25** | **OK05711W** | 3 | 3 | 3 | 3 | | | 3 | 3 |
| **26** | **OK05723W** | 1 | 1 | 1 | 1 | | | 1 | 1 |
| **27** | **OK05108** | 4 | 4 | 4 | 4 | | | 5 | 5 |
| **28** | **OK05122** | 3 | 3 | 3 | 3 | | | 3 | 3 |
| **29** | **OK05526** | 4 | 4 | 4 | 4 | | | 5 | 5 |
| **30** | **OK05134** | 2 | 2 | 2 | 2 | | | 2 | 2 |
| **31** | **OK05303** | 3 | 3 | 3 | 3 | | | 3 | 3 |
| **32** | **OK05312** | 3 | 3 | 3 | 3 | | | 3 | 3 |
| **33** | **OK05511** | 2 | 2 | 2 | 2 | | | 2 | 2 |
| **34** | **OK05204** | 2 | 2 | 2 | 2 | | | 2 | 2 |
| **35** | **GARRISON** | 2 | 2 | 2 | 2 | | | 2 | 2 |
| **36** | **OK06114** | 2 | 2 | 2 | 2 | | | 2 | 2 |
| **37** | **OK06210** | 2 | 2 | 2 | 2 | | | 2 | 2 |
| **38** | **OK06319** | 4 | 4 | 4 | 4 | | | 5 | 5 |
| **39** | **OK06318** | 3 | 3 | 3 | 3 | | | 3 | 3 |
| **40** | **OK06336** | 3 | 3 | 3 | 3 | | | 3 | 3 |
| **41** | **AGATE** | 3 | 3 | 3 | 3 | | | 3 | 3 |
| **42** | **ALLIANCE** | 3 | 3 | 3 | 3 | | | 3 | 3 |
| **43** | **ANTELOPE** | 5 | 5 | 5 | 5 | | | 5 | 5 |
| **44** | **ARAPAHOE** | 3 | 3 | 3 | 3 | | | 3 | 3 |
| **45** | **BENNETT** | 3 | 3 | 3 | 3 | | | 3 | 3 |
| **46** | **BUCKSKIN** | 2 | 2 | 2 | 2 | | | 2 | 2 |
| **47** | **CENTURA** | 2 | 2 | 2 | 2 | | | 2 | 2 |
| **48** | **CENTURK78** | 3 | 3 | 3 | 3 | | | 3 | 3 |
| **49** | **CHEYENNE** | 3 | 3 | 3 | 3 | | | 3 | 3 |
| **50** | **COLT** | 2 | 2 | 2 | 2 | | | 2 | 2 |
| **51** | **COUGAR** | 2 | 2 | 2 | 2 | | | 2 | 2 |
| **52** | **CULVER** | 2 | 2 | 2 | 2 | | | 2 | 2 |
| **53** | **GAGE** | 2 | 2 | 2 | 2 | | | 2 | 2 |
| **54** | **GOODSTREAK** | 1 | 1 | 1 | 1 | | | 1 | 1 |
| **55** | **HALLAM** | 2 | 2 | 2 | 2 | | | 2 | 2 |
| **56** | **HARRY** | 2 | 2 | 2 | 2 | | | 2 | 2 |
| **57** | **HOMESTEAD** | 2 | 2 | 2 | 2 | | | 3 | 2 |
| **58** | **INFINITY_CL** | 2 | 2 | 2 | 2 | | | 3 | 2 |
| **59** | **KHARKOF** | 3 | 3 | 3 | 3 | | | 3 | 3 |
| **60** | **MILLENNIUM** | 2 | 2 | 2 | 2 | | | 3 | 3 |
| **61** | **CAMELOT** | 3 | 3 | 3 | 3 | | | 3 | 3 |
| **62** | **OVERLAND** | 2 | 2 | 2 | 3 | | | 3 | 3 |
| **63** | **NE99495** | 3 | 3 | 3 | 3 | | | 3 | 3 |
| **64** | **NIOBRARA** | 2 | 2 | 2 | 3 | | | 3 | 3 |
| **65** | **NUPLAINS** | 3 | 3 | 3 | 3 | | | 3 | 3 |
| **66** | **PRONGHORN** | 3 | 3 | 3 | 3 | | | 3 | 3 |
| **67** | **RAWHIDE** | 2 | 2 | 2 | 3 | | | 3 | 3 |
| **68** | **REDLAND** | 3 | 3 | 3 | 3 | | | 3 | 3 |
| **69** | **SCOUT66** | 1 | 1 | 1 | 1 | | | 1 | 1 |
| **70** | **SIOUXLAND** | 3 | 3 | 4 | 3 | | | 4 | 4 |
| **71** | **TURKEY_NEBSEL** | 2 | 2 | 2 | 3 | | | 3 | 3 |
| **72** | **VISTA** | 1 | 1 | 1 | 1 | | | 1 | 1 |
| **73** | **WAHOO** | 2 | 2 | 2 | 3 | | | 3 | 3 |
| **74** | **WARRIOR** | 2 | 2 | 2 | 3 | | | 3 | 3 |
| **75** | **WESLEY** | 3 | 3 | 4 | 3 | | | 4 | 4 |
| **76** | **WICHITA** | 3 | 3 | 4 | 3 | | | 4 | 4 |
| **77** | **WINDSTAR** | 3 | 3 | 4 | 3 | | | 4 | 4 |
| **78** | **JAGGER** | 3 | 3 | 4 | 3 | | | 4 | 4 |
| **79** | **LANCER** | 2 | 2 | 2 | 3 | | | 3 | 3 |
| **80** | **SETTLER_CL** | 2 | 2 | 2 | 3 | | | 3 | 3 |
| **81** | **ANTON** | 3 | 4 | 4 | 4 | | | 4 | 4 |
| **82** | **MACE** | 3 | 4 | 4 | 4 | | | 4 | 4 |
| **83** | **JERRY** | 3 | 4 | 4 | 4 | | | 4 | 4 |
| **84** | **TAM107-R7** | 5 | 5 | 5 | 5 | | | 5 | 5 |
| **85** | **ARLIN** | 3 | 4 | 4 | 4 | | | 4 | 4 |
| **86** | **ALICE** | 2 | 2 | 2 | 3 | | | 3 | 3 |
| **87** | **DARRELL** | 2 | 2 | 2 | 3 | | | 3 | 3 |
| **88** | **EXPEDITION** | 2 | 2 | 2 | 3 | | | 3 | 3 |
| **89** | **WENDY** | 1 | 1 | 1 | 1 | | | 1 | 1 |
| **90** | **SD00111-9** | 2 | 2 | 3 | 3 | | | 3 | 3 |
| **91** | **SD01237** | 2 | 3 | 3 | 3 | | | 3 | 3 |
| **92** | **SD01058** | 3 | 4 | 4 | 4 | | | 4 | 4 |
| **93** | **SD05118** | 4 | 4 | 4 | 4 | | | 4 | 4 |
| **94** | **SD05210** | 2 | 3 | 3 | 3 | | | 3 | 3 |
| **95** | **SD05W018** | 2 | 3 | 3 | 3 | | | 3 | 3 |
| **96** | **NEKOTA** | 2 | 3 | 3 | 3 | | | 3 | 3 |
| **97** | **TANDEM** | 4 | 4 | 4 | 4 | | | 4 | 4 |
| **98** | **CRIMSON** | 4 | 4 | 4 | 4 | | | 4 | 4 |
| **99** | **ROSE** | 4 | 4 | 4 | 4 | | | 4 | 4 |
| **100** | **DAWN** | 2 | 3 | 3 | 3 | | | 3 | 3 |
| **101** | **WINOKA** | 2 | 3 | 3 | 3 | | | 3 | 3 |
| **102** | **NELL** | 5 | 5 | 5 | 5 | | | 5 | 5 |
| **103** | **RITA** | 4 | 4 | 4 | 4 | | | 4 | 4 |
| **104** | **BRONZE** | 2 | 3 | 3 | 3 | | | 3 | 3 |
| **105** | **HUME** | 2 | 3 | 3 | 3 | | | 3 | 3 |
| **106** | **GENT** | 4 | 4 | 4 | 4 | | | 4 | 4 |
| **107** | **HARDING** | 2 | 3 | 3 | 3 | | | 3 | 3 |
| **108** | **HV9W03-1551WP** | 4 | 4 | 4 | 4 | | | 4 | 4 |
| **109** | **G1878** | 4 | 4 | 4 | 4 | | | 4 | 4 |
| **110** | **HV9W03-1379R** | 2 | 3 | 3 | 3 | | | 3 | 3 |
| **111** | **HV9W03-1596R** | 4 | 4 | 4 | 4 | | | 4 | 4 |
| **112** | **HV9W05-1280R** | 4 | 4 | 4 | 4 | | | 4 | 4 |
| **113** | **HV9W06-504** | 4 | 4 | 4 | 4 | | | 4 | 4 |
| **114** | **SPARTAN** | 4 | 4 | 4 | 4 | | | 4 | 4 |
| **115** | **HV906-865** | 2 | 3 | 3 | 3 | | | 3 | 3 |
| **116** | **TARKIO** | 4 | 4 | 4 | 4 | | | 4 | 4 |
| **117** | **SMOKYHILL** | 4 | 4 | 4 | 4 | | | 4 | 4 |
| **118** | **SHOCKER** | 4 | 4 | 4 | 4 | | | 4 | 4 |
| **119** | **VONA** | 2 | 3 | 3 | 3 | | | 3 | 3 |
| **120** | **CO940610** | 2 | 3 | 3 | 3 | | | 3 | 3 |
| **121** | **AVALANCHE** | 4 | 4 | 4 | 4 | | | 4 | 4 |
| **122** | **BOND_CL** | 4 | 4 | 4 | 4 | | | 4 | 4 |
| **123** | **PLATTE** | 2 | 3 | 3 | 3 | | | 3 | 3 |
| **124** | **LINDON** | 4 | 4 | 4 | 4 | | | 4 | 4 |
| **125** | **CO03W043** | 2 | 3 | 3 | 3 | | | 3 | 3 |
| **126** | **CO03W054** | 4 | 4 | 4 | 4 | | | 4 | 4 |
| **127** | **THUNDER_CL** | 2 | 3 | 3 | 3 | | | 3 | 3 |
| **128** | **CO04025** | 4 | 4 | 4 | 4 | | | 4 | 4 |
| **129** | **CO04393** | 2 | 3 | 3 | 3 | | | 3 | 3 |
| **130** | **CO04499** | 4 | 4 | 4 | 4 | | | 4 | 4 |
| **131** | **CO04W320** | 4 | 4 | 4 | 4 | | | 4 | 4 |
| **132** | **LAMAR** | 2 | 3 | 3 | 3 | | | 3 | 3 |
| **133** | **CARSON** | 4 | 4 | 4 | 4 | | | 4 | 4 |
| **134** | **HAIL** | 4 | 4 | 4 | 4 | | | 4 | 4 |
| **135** | **SANDY** | 3 | 3 | 3 | 3 | | | 3 | 3 |
| **136** | **DUKE** | 4 | 4 | 4 | 4 | | | 4 | 4 |
| **137** | **HALT** | 3 | 3 | 3 | 3 | | | 3 | 3 |
| **138** | **HATCHER** | 3 | 3 | 3 | 3 | | | 3 | 3 |
| **139** | **PRAIRIE_RED** | 3 | 3 | 3 | 3 | | | 3 | 3 |
| **140** | **YUMAR** | 4 | 4 | 4 | 4 | | | 4 | 4 |
| **141** | **ABOVE** | 3 | 3 | 3 | 3 | | | 3 | 3 |
| **142** | **CO03064** | 3 | 3 | 3 | 3 | | | 3 | 3 |
| **143** | **BILL_BROWN** | 3 | 3 | 3 | 3 | | | 3 | 3 |
| **144** | **RIPPER** | 4 | 4 | 4 | 4 | | | 4 | 4 |
| **145** | **PROWERS** | 3 | 3 | 3 | 3 | | | 3 | 3 |
| **146** | **AKRON** | 4 | 4 | 4 | 4 | | | 4 | 4 |
| **147** | **JULES** | 4 | 4 | 4 | 4 | | | 4 | 4 |
| **148** | **YUMA** | 4 | 4 | 4 | 4 | | | 4 | 4 |
| **149** | **TAMW-101** | 4 | 4 | 4 | 4 | | | 4 | 4 |
| **150** | **TAM105** | 4 | 4 | 4 | 4 | | | 4 | 4 |
| **151** | **TAM107** | 3 | 3 | 3 | 3 | | | 3 | 3 |
| **152** | **TAM109** | 4 | 4 | 4 | 4 | | | 4 | 4 |
| **153** | **TAM110** | 4 | 4 | 4 | 4 | | | 4 | 4 |
| **154** | **TAM111** | 4 | 4 | 4 | 4 | | | 4 | 4 |
| **155** | **TAM112** | 3 | 3 | 3 | 3 | | | 3 | 3 |
| **156** | **TAM200** | 4 | 4 | 4 | 4 | | | 4 | 4 |
| **157** | **TAM202** | 3 | 3 | 3 | 3 | | | 3 | 3 |
| **158** | **TAM203** | 4 | 4 | 4 | 4 | | | 4 | 4 |
| **159** | **TAM302** | 3 | 3 | 3 | 3 | | | 3 | 3 |
| **160** | **TAM303** | 4 | 4 | 4 | 4 | | | 4 | 4 |
| **161** | **TAM304** | 5 | 5 | 5 | 5 | | | 5 | 5 |
| **162** | **TAM400** | 3 | 3 | 3 | 3 | | | 3 | 3 |
| **163** | **LOCKETT** | 5 | 5 | 5 | 5 | | | 5 | 5 |
| **164** | **STURDY** | 3 | 3 | 3 | 3 | | | 3 | 3 |
| **165** | **STURDY_2K** | 4 | 4 | 4 | 4 | | | 4 | 4 |
| **166** | **MIT** | 5 | 5 | 5 | 5 | | | 5 | 5 |
| **167** | **CAPROCK** | 3 | 3 | 3 | 3 | | | 3 | 3 |
| **168** | **TX01A5936** | 4 | 4 | 4 | 4 | | | 4 | 4 |
| **169** | **TAM401** | 3 | 3 | 3 | 3 | | | 3 | 3 |
| **170** | **TX02A0252** | 3 | 3 | 3 | 3 | | | 3 | 3 |
| **171** | **TX03A0148** | 3 | 3 | 3 | 3 | | | 3 | 3 |
| **172** | **TX03A0563** | 3 | 3 | 3 | 3 | | | 3 | 3 |
| **173** | **TX04A001246** | 3 | 3 | 3 | 3 | | | 3 | 3 |
| **174** | **TX01V5134RC-3** | 4 | 4 | 4 | 4 | | | 4 | 4 |
| **175** | **TX04M410164** | 4 | 4 | 4 | 4 | | | 4 | 4 |
| **176** | **TX04M410211** | 3 | 3 | 3 | 3 | | | 3 | 3 |
| **177** | **TX04V075080** | 4 | 4 | 4 | 4 | | | 4 | 4 |
| **178** | **TX99A0153-1** | 3 | 3 | 3 | 3 | | | 3 | 3 |
| **179** | **TX01M5009-28** | 3 | 3 | 3 | 3 | | | 3 | 3 |
| **180** | **TX00V1131** | 3 | 3 | 3 | 3 | | | 3 | 3 |
| **181** | **TX99U8618** | 3 | 3 | 3 | 3 | | | 3 | 3 |
| **182** | **TX96D1073** | 4 | 4 | 4 | 4 | | | 4 | 4 |
| **183** | **2180** | 4 | 4 | 4 | 4 | | | 4 | 4 |
| **184** | **HG-9** | 4 | 4 | 4 | 4 | | | 4 | 4 |
| **185** | **TX86A5606** | 4 | 4 | 4 | 4 | | | 4 | 4 |
| **186** | **TX86A6880** | 3 | 3 | 3 | 3 | | | 3 | 3 |
| **187** | **TX86A8072** | 4 | 4 | 4 | 4 | | | 4 | 4 |
| **188** | **CREST** | 4 | 4 | 4 | 4 | | | 4 | 4 |
| **189** | **ROSEBUD** | 4 | 4 | 4 | 4 | | | 4 | 4 |
| **190** | **JUDITH** | 3 | 3 | 3 | 3 | | | 3 | 3 |
| **191** | **MT85200** | 4 | 4 | 4 | 4 | | | 4 | 4 |
| **192** | **NUSKY** | 3 | 3 | 3 | 3 | | | 3 | 3 |
| **193** | **MT9513** | 4 | 4 | 4 | 4 | | | 4 | 4 |
| **194** | **MT9904** | 3 | 3 | 3 | 3 | | | 3 | 3 |
| **195** | **MT9982** | 3 | 3 | 3 | 3 | | | 3 | 3 |
| **196** | **GENOU** | 4 | 4 | 4 | 4 | | | 4 | 4 |
| **197** | **NORRIS** | 4 | 4 | 4 | 4 | | | 4 | 4 |
| **198** | **YELLOWSTONE** | 3 | 3 | 3 | 3 | | | 3 | 3 |
| **199** | **MT0495** | 4 | 4 | 4 | 4 | | | 4 | 4 |
| **200** | **MTS0531** | 4 | 4 | 4 | 4 | | | 4 | 4 |
| **201** | **DECADE** | 3 | 3 | 3 | 3 | | | 3 | 3 |
| **202** | **MT06103** | 4 | 4 | 4 | 4 | | | 4 | 4 |
| **203** | **JUDEE** | 3 | 3 | 3 | 3 | | | 3 | 3 |
| **204** | **LAKIN** | 3 | 3 | 3 | 3 | | | 3 | 3 |
| **205** | **STANTON** | 4 | 4 | 4 | 4 | | | 4 | 4 |
| **206** | **TREGO** | 3 | 3 | 3 | 3 | | | 3 | 3 |
| **207** | **KARL_92** | 4 | 4 | 4 | 4 | | | 4 | 4 |
| **208** | **DODGE** | 3 | 3 | 3 | 3 | | | 3 | 3 |
| **209** | **NORKAN** | 3 | 3 | 3 | 3 | | | 3 | 3 |
| **210** | **CHENEY** | 3 | 3 | 3 | 3 | | | 3 | 3 |
| **211** | **NEWTON** | 3 | 3 | 3 | 3 | | | 3 | 3 |
| **212** | **LARNED** | 2 | 2 | 2 | 2 | | | 2 | 2 |
| **213** | **PARKER76** | 3 | 3 | 3 | 3 | | | 3 | 3 |
| **214** | **KIRWIN** | 4 | 4 | 4 | 4 | | | 4 | 4 |
| **215** | **SAGE** | 3 | 3 | 3 | 3 | | | 3 | 3 |
| **216** | **TRISON** | 4 | 4 | 4 | 4 | | | 4 | 4 |
| **217** | **EAGLE** | 1 | 1 | 1 | 1 | | | 1 | 1 |
| **218** | **SHAWNEE** | 4 | 4 | 4 | 4 | | | 4 | 4 |
| **219** | **PARKER** | 3 | 3 | 3 | 3 | | | 3 | 3 |
| **220** | **KAW61** | 4 | 4 | 4 | 4 | | | 4 | 4 |
| **221** | **TASCOSA** | 4 | 4 | 4 | 4 | | | 4 | 4 |
| **222** | **BISON** | 4 | 4 | 4 | 4 | | | 4 | 4 |
| **223** | **KIOWA** | 4 | 4 | 4 | 4 | | | 4 | 4 |
| **224** | **COMANCHE** | 3 | 3 | 3 | 3 | | | 3 | 3 |
| **225** | **BAKERS_WHITE** | 4 | 4 | 4 | 4 | | | 4 | 4 |
| **226** | **BURCHETT** | 3 | 3 | 3 | 3 | | | 3 | 3 |
| **227** | **CUTTER** | 3 | 3 | 3 | 3 | | | 3 | 3 |
| **228** | **DUMAS** | 4 | 4 | 4 | 4 | | | 4 | 4 |
| **229** | **HONDO** | 4 | 4 | 4 | 4 | | | 4 | 4 |
| **230** | **JAGALENE** | 3 | 3 | 3 | 3 | | | 3 | 3 |
| **231** | **LONGHORN** | 3 | 3 | 3 | 3 | | | 3 | 3 |
| **232** | **NEOSHO** | 4 | 4 | 4 | 4 | | | 4 | 4 |
| **233** | **OGALLALA** | 4 | 4 | 4 | 4 | | | 4 | 4 |
| **234** | **POSTROCK** | 3 | 3 | 3 | 3 | | | 3 | 3 |
| **235** | **THUNDERBOLT** | 3 | 3 | 3 | 3 | | | 3 | 3 |
| **236** | **W04-417** | 4 | 4 | 4 | 4 | | | 4 | 4 |
| **237** | **NUFRONTIER** | 3 | 3 | 3 | 3 | | | 3 | 3 |
| **238** | **NUHORIZON** | 3 | 3 | 3 | 3 | | | 3 | 3 |
| **239** | **ONAGA** | 3 | 3 | 3 | 3 | | | 3 | 3 |
| **240** | **RONL** | 3 | 3 | 3 | 3 | | | 3 | 3 |
| **241** | **2145** | 3 | 3 | 3 | 3 | | | 3 | 3 |
| **242** | **HEYNE** | 3 | 3 | 3 | 3 | | | 3 | 3 |
| **243** | **KS00F5-20-3** | 4 | 4 | 4 | 4 | | | 4 | 5 |
| **244** | **OVERLEY** | 4 | 4 | 4 | 4 | | | 5 | 5 |
| **245** | **FULLER** | 3 | 3 | 3 | 3 | | | 3 | 3 |
| **246** | **COSSACK** | 4 | 4 | 5 | 4 | | | 5 | 5 |
| **247** | **ENHANCER** | 3 | 3 | 3 | 3 | | | 3 | 3 |
| **248** | **SANTA_FE** | 4 | 4 | 5 | 4 | | | 5 | 5 |
| **249** | **VENANGO** | 4 | 4 | 5 | 4 | | | 5 | 5 |
| **250** | **WB411W** | 3 | 3 | 3 | 3 | | | 3 | 3 |
| **251** | **KEOTA** | 3 | 3 | 3 | 3 | | | 3 | 3 |
| **252** | **TX05A001822** | 4 | 4 | 5 | 4 | | | 5 | 5 |
| **253** | **TX06A001263** | 3 | 3 | 3 | 3 | | | 3 | 3 |
| **254** | **TX06A001132** | 3 | 3 | 3 | 3 | | | 3 | 3 |
| **255** | **TX06A001281** | 4 | 4 | 5 | 4 | | | 5 | 5 |
| **256** | **TX06A001386** | 3 | 3 | 3 | 3 | | | 3 | 3 |
| **257** | **TX05V7259** | 4 | 5 | 5 | 5 | | | 5 | 5 |
| **258** | **TX05V7269** | 3 | 3 | 3 | 3 | | | 3 | 3 |
| **259** | **TX05A001188** | 3 | 3 | 3 | 3 | | | 3 | 3 |
| **260** | **TX07A001279** | 4 | 5 | 5 | 5 | | | 5 | 5 |
| **261** | **TX07A001318** | 3 | 3 | 3 | 3 | | | 3 | 3 |
| **262** | **TX07A001420** | 1 | 1 | 1 | 1 | | | 1 | 1 |
| **263** | **TX06V7266** | 3 | 3 | 3 | 3 | | | 3 | 3 |
| **264** | **OK1067071** | 3 | 3 | 3 | 3 | | | 3 | 3 |
| **265** | **OK1067274** | 4 | 5 | 5 | 5 | | | 5 | 5 |
| **266** | **OK1068002** | 3 | 3 | 3 | 3 | | | 3 | 3 |
| **267** | **OK1068009** | 5 | 5 | 5 | 5 | | | 5 | 5 |
| **268** | **OK1068026** | 3 | 3 | 3 | 3 | | | 3 | 3 |
| **269** | **OK1068112** | 1 | 1 | 1 | 1 | | | 1 | 1 |
| **270** | **OK1070275** | 3 | 3 | 3 | 3 | | | 3 | 3 |
| **271** | **OK1070267** | 5 | 5 | 5 | 5 | | | 5 | 5 |
| **272** | **OK09634** | 5 | 5 | 5 | 5 | | | 5 | 5 |
| **273** | **OK10119** | 5 | 5 | 5 | 5 | | | 5 | 5 |
| **274** | **GALLAGHER** | 3 | 3 | 3 | 3 | | | 3 | 3 |
| **275** | **OK07231** | 3 | 3 | 3 | 3 | | | 3 | 3 |
| **276** | **OK07S117** | 3 | 3 | 3 | 3 | | | 3 | 3 |
| **277** | **OK08328** | 5 | 5 | 5 | 5 | | | 5 | 5 |
| **278** | **BIG_SKY** | 3 | 3 | 3 | 3 | | | 3 | 3 |
| **279** | **DANBY** | 3 | 3 | 3 | 3 | | | 3 | 3 |
| **280** | **E2041** | 3 | 3 | 3 | 3 | | | 3 | 3 |
| **281** | **DENALI** | 5 | 5 | 5 | 5 | | | 5 | 5 |
| **282** | **CO050337-2** | 3 | 3 | 3 | 3 | | | 3 | 3 |
| **283** | **BYRD** | 5 | 5 | 5 | 5 | | | 5 | 5 |
| **284** | **CO07W245** | 3 | 3 | 3 | 3 | | | 3 | 3 |
| **285** | **MCGILL** | 3 | 3 | 3 | 3 | | | 3 | 3 |
| **286** | **NE02558** | 3 | 3 | 3 | 3 | | | 3 | 3 |
| **287** | **NW03666** | 5 | 5 | 5 | 5 | | | 5 | 5 |
| **288** | **NE04490** | 1 | 1 | 1 | 1 | | | 1 | 1 |
| **289** | **NE05430** | 3 | 3 | 3 | 3 | | | 3 | 3 |
| **290** | **NE05496** | 3 | 3 | 3 | 3 | | | 3 | 3 |
| **291** | **NE05548** | 5 | 5 | 5 | 5 | | | 5 | 5 |
| **292** | **NE06545** | 5 | 5 | 5 | 5 | | | 5 | 5 |
| **293** | **NE06607** | 5 | 5 | 5 | 5 | | | 5 | 5 |
| **294** | **ROBIDOUX** | 1 | 1 | 1 | 1 | | | 1 | 1 |
| **295** | **NI06736** | 3 | 3 | 3 | 3 | | | 3 | 3 |
| **296** | **NI06737** | 5 | 5 | 5 | 5 | | | 5 | 5 |
| **297** | **NI07703** | 3 | 3 | 3 | 3 | | | 3 | 3 |
| **298** | **NI08707** | 3 | 3 | 3 | 3 | | | 3 | 3 |
| **299** | **NI08708** | 3 | 3 | 3 | 3 | | | 3 | 3 |
| **Mean** |  | 3.15 | 3.24 | 3.27 | 3.28 | | | 3.33 | 3.33 |
